# Assembly and Comparative Analysis of Complete Mitochondrial Genome Sequence of Endangered Medicinal Plant *Trichopus zeylanicus*

**DOI:** 10.3390/cimb47070553

**Published:** 2025-07-16

**Authors:** Biju Vadakkemukadiyil Chellappan, P. R. Shidhi, Anu Sasi, Rashid Ismael Hag Ibrahim, Hamad Abu Zahra

**Affiliations:** 1 Department of Biological Sciences, College of Science, King Faisal University, P.O. Box 420, Al-Ahsa 31982, Saudi Arabia; ribrahim@kfu.edu.sa (R.I.H.I.); habuzahra@kfu.edu.sa (H.A.Z.); 2 Department of Zoology, University of Kerala, Kariyavattom Campus, Thiruvananthapuram 695581, Kerala, India; shidhidcb@keralauniversity.ac.in; 3 Department of Computational Biology and Bioinformatics, University of Kerala, Kariyavattom Campus, Thiruvananthapuram 695581, Kerala, India; anusasi2025@keralauniversity.ac.in

**Keywords:** genetic diversity, comparative genomics, codon usage bias, mitochondrial repetitive elements

## Abstract

Plant mitochondrial genomes exhibit extensive size variability and structural complexity. Here, we report the complete mitochondrial genome of *Trichopus zeylanicus*, an endemic medicinal plant from the Western Ghats. The mitochondrial genome was assembled using a combination of Illumina short-read and PacBio long-read sequencing technologies, followed by extensive annotation and comparative analysis. The circular mitogenome spans 709,127 bp with a GC content of 46%, encoding 32 protein-coding genes, 17 tRNAs, and three rRNAs. Comparative analysis with other monocot mitochondrial genomes revealed conserved gene clusters but also significant lineage-specific rearrangements. Despite genome size similarities, *T. zeylanicus* displayed marked divergence in gene order, suggesting that genome size does not necessarily correlate with structural conservation. The genome contains 6.7% chloroplast-derived sequences and 324 predicted RNA-editing sites, predominantly in the first and second codon positions. Phylogenetic analysis based on mitochondrial genes placed *T. zeylanicus* as a distinct lineage within Dioscoreales, supporting its evolutionary uniqueness. This work provides the first mitogenomic resource for Dioscoreales and advances our understanding of mitochondrial diversity and evolution in monocots.

## 1. Introduction

Plant mitochondrial genomes exhibit remarkable diversity and complexity compared to their animal counterparts [1]. Their sizes vary significantly from 66 kb to 11,000 kb and can exist in circular, linear, or multichromosomal structures [2,3,4]. Despite their size, they contain relatively few genes, with most of the DNA consisting of introns, repeats, and non-coding regions [5]. This complexity is further amplified by their dynamic nature, which includes frequent structural rearrangements, intragenomic recombination facilitated by large repeats, and the incorporation of foreign DNA through horizontal gene transfer from nuclear and chloroplast genomes [4,5]. These features make plant mitochondrial genomes highly unique and integral to understanding plant evolution [6,7].

Plant mitochondria rely on post-transcriptional RNA editing to ensure the proper functioning of mitochondrial genes. RNA editing in plant mitochondria converts specific cytidines to uridines in mRNA, with occasional reverse conversions [8]. This process affects nearly all mRNAs and can significantly alter the encoded proteins [9]. The recognition of RNA-editing sites predominantly relies on the 5’ flanking RNA sequence rather than its secondary structure [10]. The editing process is crucial for producing functional proteins and may be required for proper RNA processing, including trans splicing [11]. RNA editing has implications for cytoplasmic male sterility, a significant characteristic in plant breeding [12]. The identification of RNA-editing sites is made possible through genome sequencing, which allows for comparisons between the DNA template and the resulting RNA transcripts, allowing the identification of precise nucleotide modifications. Understanding RNA editing in plant mitochondria not only sheds light on the regulation of mitochondrial gene expression but also on the evolution of this unique mechanism across plant species.

*Trichopus zeylanicus*, a medicinal plant endemic to the Western Ghats of India and Sri Lanka, is garnering increasing interest among researchers [13]. *T. zeylanicus*, commonly referred to as “Arogyapacha”, is renowned for its anti-fatigue properties among indigenous populations. In addition to its anti-fatigue effects, this plant exhibits a diverse array of pharmacological properties, including antimicrobial, antioxidant, aphrodisiac, antistress, immunomodulatory, anti-inflammatory, anti-tumor, anti-ulcer, hepatoprotective, anti-hyperlipidemic, and anti-diabetic activities [14,15,16,17,18,19,20]. Despite its pharmacological importance, *T. zeylanicus* is currently classified under the order Dioscoreales and family Dioscoreaceae according to the Angiosperm Phylogeny Group IV system [21]. However, its familial position has been debated, as it has repeatedly been reassigned [22,23,24]. Recently, the nuclear and chloroplast genomes of *T. zeylanicus* were released, which paved the way for studying the secondary metabolic biosynthesis pathways in this medicinal plant [25,26]. Nevertheless, the characteristics of its mitochondrial genome remain largely unexplored, potentially containing critical insights into its evolutionary adaptation and phylogenetic placement within the plant kingdom.

This study presents the first report and comprehensive analysis of the mitochondrial genome of *T. zeylanicus*. The entire genome was annotated to enable us to analyze its genomic composition, gene content, repetitive sequences, chloroplast genome fragment migration, and RNA-editing sites, and a phylogenetic study with closely related species was conducted. This will serve as a significant resource for future research on mitogenomes within the order Dioscoreales, as it is the first thoroughly described mitogenome in this order.

## 2. Materials and Methods

### 2.1. Plant Collection and Genomic DNA Extraction

Specimens of *T. zeylanicus* were collected from the Agastya Hills region in Trivandrum, Kerala, India, and used in this study. The specimens (Voucher KUBH5870) were deposited in the Herbarium of the Department of Botany, University of Kerala, India. Genomic DNA was extracted from young leaf tissues following the CTAB protocol as described by Healey et al. (2014) [27].

### 2.2. Library Preparation, Sequencing, and Mitochondrial Genome Assembly

Approximately 1 µg of purified genomic DNA was fragmented to an average size of 300 bp using a Covaris S220 Focused-ultrasonicator (Covaris, Woburn, MA, USA). DNA libraries were constructed using the NEBNext Ultra II DNA Library Prep Kit (New England Biolabs, Ipswich, MA, USA) according to the manufacturer’s instructions. An Illumina HiSeq 2500 (2 × 100 bp paired-end mode; Illumina, San Diego, CA, USA) was utilized for sequencing. For long-read sequencing, about 30 µg of DNA was fragmented using a Covaris g-TUBE (Covaris, Woburn, MA, USA) and processed into single-molecule real-time (SMRT) bell templates ranging from 15 to 50 kb following Pacific Biosciences’ guidelines (Pacific Biosciences, Menlo Park, CA, USA). The PacBio Sequel platform, employing the P6-C4 chemistry across five SMRT cells, generated the long-read data.

Raw Illumina sequencing reads were pre-processed using AdapterRemoval v2 to excise residual adapter sequences, trim low-quality bases (Phred score < 20), and remove reads shorter than 50 bp [28]. PacBio polymerase reads were quality-filtered using the SMRT Analysis suite by excluding reads with an average accuracy below Q20 (corresponding to a predicted error rate > 1%), discarding polymerase reads shorter than 100 bp and subreads less than 500 bp. Because sequencing was performed on total leaf-derived DNA, both Illumina and PacBio reads were initially mapped against the nuclear and chloroplast genomes of *T. zeylanicus* to eliminate non-mitochondrial sequences [25,26]. The resulting mitochondrial-enriched short reads were retained for mitochondrial genome assembly. Before assembly, PacBio reads were error-corrected with high-quality Illumina reads using Proovread v2.12 [29]. Corrected long reads were subsequently assembled into a single contiguous sequence using Canu v1.8 [30]. The completeness and coverage depth of the final assembly were assessed by aligning all Illumina reads with Bowtie and PacBio reads using Blasr v5.3..

### 2.3. Mitochondrial Genome Annotation

Protein-coding genes (PCGs), ribosomal RNA (rRNA), and transfer RNA (tRNA) genes were annotated utilizing the GEseq webserver [31]. Gene coordinates, including start and end positions, were manually validated, and refined through BLAST v2.16.0+ searches against reference mitochondrial genomes. A circular mitochondrial genome map was generated using the OrganellarGenomeDRAW (OGDRAW) software v1.3.1 [32]. The whole mitochondrial genome data of *T. zeylanicus* has been submitted to GenBank with the accession number OR830326. An assessment of nucleotide composition skewness was performed utilizing the following equations: AT skew, determined as (A − T)/(A + T), and GC skew, determined as (G − C)/(G + C) [33].

### 2.4. Identification of Repetitive Elements

Palindromic, forward, reverse, and complementary repeats were identified using REPuter v2.74 [34]. The minimum repeat size examined in the present study was 20 base pairs (bp). The detection of simple sequence repeats (SSRs) was executed using the MISA software v2.1 [35].

### 2.5. Identification of RNA Editing Sites

The identification of RNA-editing sites in the protein-coding genes of the *T. zeylanicus* mitochondrial genome was performed with PREPACT 3.0 [36], employing a cut-off value of E = 0.00001 to ensure high precision in the predictions.

### 2.6. Chloroplast and Mitochondrial Genome Migration

To identify interorganellar gene transfers from the chloroplast genome to the mitochondrial genome, the mitochondrial genome was mapped against the chloroplast genome using the BLASTN tool with default parameters. The gene transfer segments between the organelle genomes were visualized using the Circos package v0.69-3 [37].

### 2.7. Phylogenetic Analysis

A phylogenetic tree was constructed to determine the evolutionary placement of *T. zeylanicus*, utilizing six mitochondrial genes from 19 species. The GenBank accession numbers for all the genes used are listed in Supplementary Table S7. Phylogenetic analysis was performed using the maximum likelihood (ML) method, implemented in PhyML v3.0, with 1000 bootstrap replicates to assess statistical support for the tree topology [38,39].

## 3. Results and Discussion

### 3.1. The Structure, Organization, and Composition of the T. zeylanicus Mitogenome

**Figure 1:**
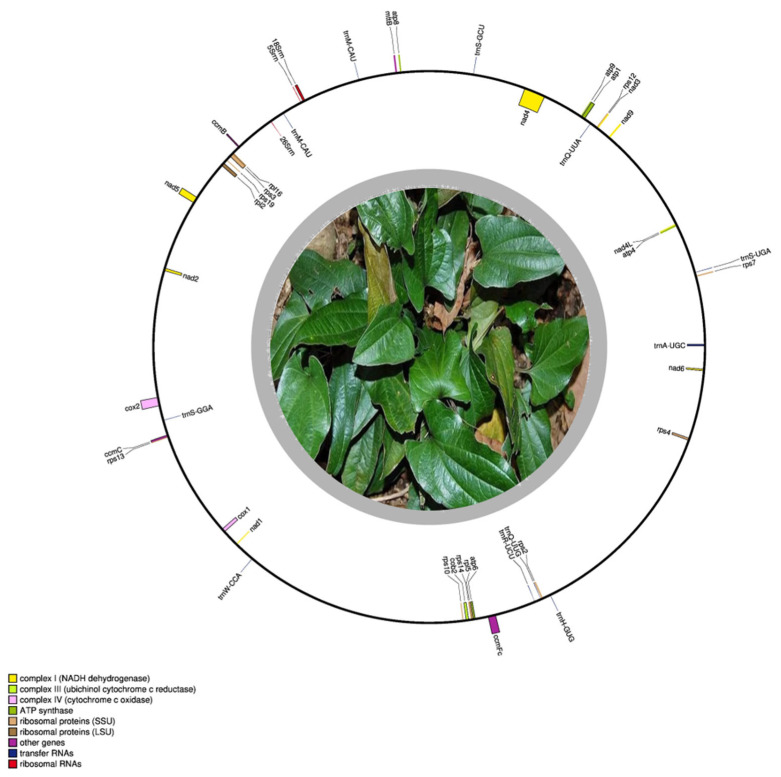
Circular visualization of the mitochondrial genome of *Trichopus zeylanicus*. Genes transcribed clockwise are labeled outside the circle, while those transcribed counterclockwise are labeled inside the circle. Different functional categories are represented by distinct colors. The central photograph depicts the characteristic morphology of *T. zeylanicus*, showing its broad, dark green leaves, as observed in its natural habitat in the Western Ghats.

This study presents the first complete mitochondrial genome of *T. zeylanicus*, a medicinally important and phylogenetically unique member of the order Dioscoreales (Pushpangadan P., 1988 [13]; Caddick et al., 2002 [40]). The mitochondrial genome of *T. zeylanicus* spans 709,127 bp in size with a GC content of 46% (Figure 1). The nucleotide composition is as follows: adenine (A) 27.19%, thymine (T) 26.82%, cytosine (C) 22.92%, and guanine (G) 23.06% This results in positive AT skew (0.006941) and GC skew (0.002986), indicating a mild predominance of adenine and guanine (Figure 1 and Figure S1). The comparative analysis indicates that the mitochondrial genome size of *T. zeylanicus* is within the expected range for angiosperms, larger than that of *Asparagus officinalis* (492,062 bp) but smaller than that of *Phoenix dactylifera* (715,001 bp). Its GC content aligns closely with that of related species, including *Cocos nucifera* (45.5%), *A. officinalis* (45.9%), and *Pandanus odorifer* (45.7%) (Table 1) [41,42].

**Table 1:** Comparative analysis of mitochondrial genome features across selected plant species related to *Trichopus zeylanicus*.

Species	GenBank Accession Number	Genome Size (bp)	GC Content (%)	Repetitive Sequence %	Chloroplast-Derived Sequences (%)	Genes			RNA-Editing Sites in PCGs *
						PCGs *	tRNAs	rRNAs	
*Trichopus zeylanicus*	OR830326	709,127	45.9	0.69	6.7	32	17	324	324
*Cocos nucifera*	NC_031696.1	678,653	45.5	17.26	5.07	72	23	734	734
*Phoenix dactylifera*	NC_016740.1	715,001	45.1	1.6	10.3	38	30	491	491
*Asparagus officinalis*	NC_053642.1	492,062	45.9	5.7	4.11	36	17	810	810
*Pandanus odorifer*	NC_080521.1	330,962	45.7	1.3	5.2	32	6	325	325

* Protein-coding genes.

### 3.2. Gene Features of the Mitochondrial Genome of T. zeylanicus

The mitochondrial genome of *T. zeylanicus* contains a total of 52 annotated genes, comprising 32 protein-coding genes (PCGs), 17 transfer RNAs (tRNAs), and three ribosomal RNAs (rRNAs) (Table 2 and Table S1). The distribution and classification of these genes align well with the typical architecture of angiosperm mitogenomes, where a conserved core of respiratory and ribosomal genes is maintained despite frequent rearrangements and size variation [43,44].

**Table 2:** Genes present in the mitochondrial genome of *Trichopus zeylanicus*.

Group of Genes	Gene Names
Complex I (NADH dehydrogenase)	*nad1*, *nad2*(2), *nad3*, *nad4*(3), *nad4l*, *nad5*, *nad6*, *nad9*
Complex III (ubiquinol cytochrome c reductase)	*cob2*|*cob*
Complex IV (cytochrome c oxidase)	*cox1*, *cox2*(2)
Complex V (ATP synthase)	*atp1*, *atp4*, *atp6*, *atp8*, *atp9*
Cytochrome c biogenesis	*ccmB*, *ccmc*, *ccmfc*(5)
Ribosomal proteins (SSU)	*rps2*(1), *rps3*, *rps4*, *rps7*, *rps10*, *rps12*, *rps13*, *rps14*, *rps19*
Ribosomal proteins (LSU)	*rpl2*, *rpl5*, *rpl16*
Transport membrane protein	*mttB*(1)
Ribosomal RNAs	*rrn26*, *rrn5*, *rrn18*
Transfer RNAs	*trnA-UGC*(2), *trnC-GCA*, *trnH-GUG*, *trnM-CAU*, *trnN-GUU*, *trnQ-UUA*(1), *trnQ-UUG*, *trnR-UCU*(1), *trnS-GCU*, *trnS-GGA*, *trnS-UGA*(1), *trnW-CCA*, *trnY-GUA*

The number of introns in each gene is indicated in brackets.

The 32 PCGs were grouped into nine major functional categories: NADH dehydrogenase complex (Complex I) including *nad1*, *nad2*, *nad3*, *nad4*, *nad4L*, *nad5*, *nad6*, and *nad9*; cytochrome c oxidase (Complex IV) with *cox1* and cox2; ATP synthase (Complex V) with *atp1*, *atp4*, *atp6*, *atp8*, and *atp9*; ubiquinol cytochrome c reductase (Complex III) represented by cob; and cytochrome c biogenesis, including *ccmB*, *ccmC*, and *ccmFc*. In addition, nine small-subunit ribosomal protein genes (*rps2*, *rps3*, *rps4*, *rps7*, *rps10*, *rps12*, *rps13*, *rps14*, and rps19) and three large-subunit ribosomal protein genes (*rpl2*, *rpl5*, *rpl16*) were identified, along with one transport membrane protein gene (*mttB*). Collectively, these genes represent the conserved backbone necessary for mitochondrial bioenergetics, translation, and gene expression (Table 2).

The total length of the protein-coding region is 38,310 bp, accounting for 5.4% of the mitochondrial genome (Table S1). The nucleotide composition within coding sequences shows a moderate AT-rich bias, with 27.54% adenine, 30.50% thymine, 20.68% cytosine, and 21.28% guanine, resulting in an overall AT content of 58.04%. Among the PCGs, 19 genes exhibited a negative AT skew, while 21 showed a positive GC skew, indicating strand-specific compositional asymmetry, a feature commonly observed in plant mitogenomes [33].

Thirteen unique transfer RNAs (tRNAs) were identified inside the mitochondrial genome, of which four of these tRNAs (*trnM-CAU*, *trnN-GUU*, *trnA-UGC,* and *trnY-GUA*) existed in duplicate (Table 2). Four transfer RNAs were identified to have introns, of which trnA-UGC possessed two introns, and the other three transfer RNAs (*trnS-UGA*, *trnQ-UUA*, and *trnR-UCU*) possessed one intron (Table S1). The total length of the transfer RNA (tRNA) was established to be 2536 base pairs (bp), constituting 0.35% of the mitochondrial genome (Table S1). Three ribosomal RNAs (rrn26, rrn5, rrn18) were found in the genome, with a total length of 1154 base pairs (bp), constituting 0.16% of the genome. When compared with closely related monocot mitogenomes, *T. zeylanicus* displays both shared and unique features (Table 1). Its PCG and tRNA gene counts (32 and 17, respectively) match those of *P. odorifer* and are slightly lower than those of *P. dactylifera* (38 PCGs, 30 tRNAs) and *C. nucifera* (72 PCGs, 23 tRNAs) [41,42,45]. This suggests either gene loss or relocation to the nuclear genome over evolutionary time. The variation in gene content and structure, particularly the number of introns and duplicated tRNAs, highlights lineage-specific evolution among monocots. Notably, *T. zeylanicus* (Dioscoreaceae) differs from other families such as Pandanaceae (*P. odorifer*) and Arecaceae (*P. dactylifera*), which display distinct intron patterns and tRNA repertoires, emphasizing the evolutionary divergence within these lineages.

### 3.3. Repetitive Sequence Analysis

**Figure 2:**
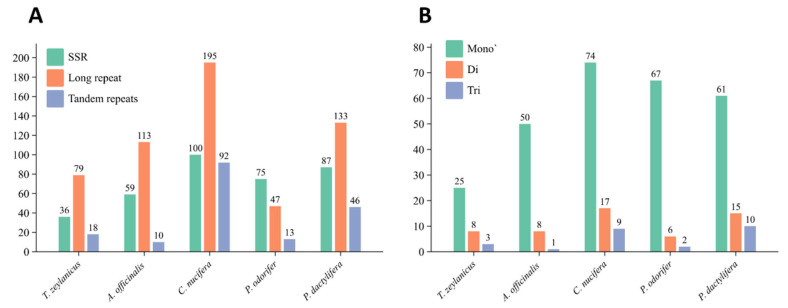
Repetitive sequences in the mitochondrial genome of *Trichopus zeylanicus* and its closely related species. (**A**). The distribution of simple sequence repeats (SSRs), long repeats, and tandem repeats across the species. The number of different types of repeats in each species is shown above the respective bars. (**B**). The distribution of different types of simple sequence repeats (SSRs) in the mitochondrial genome of *T. zeylanicus*. The numbers above the bars represent the copy number of each repeat.

The mitochondrial genome of *T. zeylanicus* exhibits a moderate abundance of repetitive DNA elements, which play a crucial role in genome structural dynamics and evolution. In total, 36 simple sequence repeats (SSRs) were identified, comprising 25 mononucleotide, 8 dinucleotide, and 3 trinucleotide repeats (Figure 2A,B, Table S2). The mononucleotide repeats were predominantly composed of A/T motifs, a common feature in plant mitogenomes that reflects the AT-rich nature of non-coding regions [5,46]. The length of the repetitive sequences varied from 9 to 14 base pairs. *T. zeylanicus* contained the lowest number of SSRs, whereas *C. nucifera* had the highest (Figure 2A). This variation may correlate with genome plasticity and the extent of repeat-mediated recombination across species (Figure 2A, Table S2). In addition to SSRs, 79 long repeat sequences were identified in the *T. zeylanicus* mitogenome (Table S3). These included 39 forward repeats and 40 palindromic repeats, while no reverse or complementary repeats were detected. The length of these repeats ranged from 106 to 1162 bp and collectively accounted for 0.52% of the mitochondrial genome. This repeat content is lower than that found in *C. nucifera* (which has the highest number of long repeats, including repeats up to 19,212 bp) and also lower than in *A. officinalis*, which contains long repeats up to 12,348 bp. This suggests that *T. zeylanicus* possesses a relatively compact repeat architecture, which may partially explain the observed level of genome rearrangement and structural divergence [6,47].

Tandem repeats, also known as satellite DNA, were additionally detected using specialized algorithms. A total of 18 perfect tandem repeats were identified, with lengths ranging from 14 to 27 bp, and repeat copy numbers between 2 and 3 (Table 3). These repeats accounted for only 0.11% of the mitogenome, further supporting the compact repeat landscape in *T. zeylanicus*. Among the analyzed species, *C. nucifera* again showed the highest number of tandem repeats (92), while *A. officinalis* had the lowest (13). The relatively low number of tandem and long repeats in *T. zeylanicus* may indicate reduced repeat-mediated recombination compared to more structurally dynamic mitogenomes, although it still exhibits sufficient repeat content to support limited intramolecular recombination and rearrangement events [5].

Despite having fewer repetitive elements than many other monocot species, *T. zeylanicus* displays clear signs of genomic rearrangement, as evidenced by its disrupted gene order compared to more structurally stable mitogenomes like *P. dactylifera* (Figure S2). This paradox, low repeat content but high rearrangement, suggests that even modest levels of repeats, particularly palindromic and forward repeats, may be sufficient to mediate genome isomerization and contribute to structural novelty [6]. In addition to repeat-mediated recombination, other factors may drive structural rearrangements in plant mitogenomes, including the activity of mobile genetic elements (e.g., transposons), double-strand break repair via non-homologous end joining (NHEJ), and homologous recombination via small dispersed repeats [47,48,49]. Moreover, oxidative stress and DNA damage in metabolically active tissues such as leaves may trigger genome instability [8]. These mechanisms, in conjunction with even modest repeat content, may explain the high structural plasticity observed in *T. zeylanicus*.

### 3.4. RNA-Editing Site Prediction

**Figure 3:**
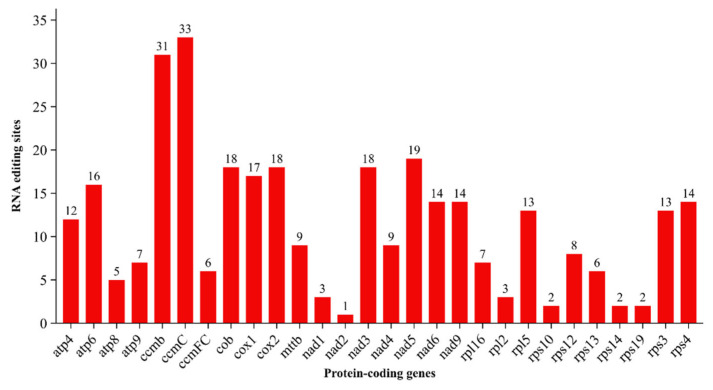
The distribution of RNA-editing sites across the 29 protein-coding genes in the mitochondrial genome of *T. zeylanicus*. The numbers above each batch represent the number of RNA-editing sites in the respective genes.

RNA editing is essential for gene expression in the mitochondrial and chloroplast genomes of all angiosperms. A total of 324 RNA-editing sites were predicted across 29 protein-coding genes (PCGs) in the mitochondrial genome of *T. zeylanicus*, using the PREPACT 3.0 platform with stringent criteria (E-value < 1 × 10^−5^) (Figure 3, Table S4). All predicted editing events involved C-to-U conversions, a canonical editing type in angiosperm mitochondria [50,51]. The distribution of editing sites revealed a positional bias, with 33% (107 sites) located in the first codon position and 67% (217 sites) in the second position. Notably, no RNA-editing events were observed in the third codon position, which is consistent with prior findings in other plant mitochondrial genomes, where editing typically targets sites that result in amino acid changes, rather than silent mutations [51]. Among the PCGs, ccmC had the highest number of editing sites (33), followed closely by ccmB (31) and nad5 (19), reflecting the intensive post-transcriptional regulation of genes involved in cytochrome c biogenesis and Complex I assembly, both of which are critical for mitochondrial respiration and energy production (Figure 3). In contrast, genes like nad2 contained only a single editing site, and nad4L, rps2, and rps7 showed no predicted RNA editing, indicating gene-specific variation in editing dependency. Such variation has also been observed in other plant species and may reflect evolutionary constraints on protein function or structural stability (Mower 2020 [43]). Amino acid conversions resulting from RNA-editing events showed a clear preference for hydrophilic-to-hydrophobic substitutions. Specifically, 182 editing events (56.17%) changed hydrophilic amino acids to hydrophobic ones, whereas 32 events (9.87%) occurred in the opposite direction. Additionally, 88 editing events (27.16%) maintained hydrophobic residues, and 41 (12.6%) preserved hydrophilic residues, indicating that many edits may function to fine-tune protein polarity and membrane affinity (Table S4). A single editing event produced a premature stop codon in the atp9 gene, suggesting a potential mechanism for gene regulation or pseudogene evolution (Table S4). These observations align with established knowledge that RNA editing in plant mitochondria not only restores conserved amino acids but can also affect protein hydrophobicity and functional domains [50,52]. The total number of editing sites in *T. zeylanicus* (324) is moderate when compared to other monocots such as *C. nucifera* (734 sites), *A. officinalis* (810), and *P. dactylifera* (491). This suggests either a reduced dependence on RNA editing or potentially greater genomic coding accuracy at the DNA level in *T. zeylanicus*. It may also reflect evolutionary streamlining in editing machinery or the selective retention of edited sites critical for mitochondrial function [43,44].

### 3.5. Codon Usage Analysis

**Figure 4:**
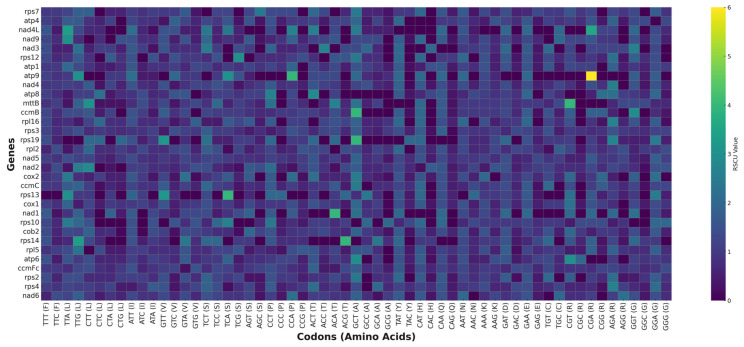
Codon usage analysis of the mitochondrial genome of *Trichopus zeylanicus*. A heatmap of relative synonymous codon usage (RSCU) values across mitochondrial protein-coding genes of *Trichopus zeylanicus*. Each row represents an individual mitochondrial gene, while each column corresponds to a specific codon labeled with its associated amino acid. The color gradient reflects the RSCU values, ranging from low (deep purple) to high (bright yellow), with values above 1.0 indicating preferred codon usage.

In the mitochondrial genome of *T. zeylanicus*, most protein-coding genes (PCGs) begin with the start codon AUG and end with the stop codon UAA, UAG, or UGA. Our analysis of codon usage indicates that AUG (Methionine), CAA (Glutamine), GAC (Aspartic acid), and GGA (Glycine) have the highest frequency among codons, whereas TGC (Cysteine), TAT (Tyrosine), and CAG (Glutamine) are noted for their lowest frequency in the protein-coding genes (PCGs) of the mitogenome (Figure 4, Table S5). The assessment of codon usage bias entailed the computation of relative synonymous codon usage (RSCU). The RSCU analysis showed that all codons were represented in the protein-coding genes. The mitogenome of *T. zeylanicus* comprises 8238 codons, allocated among 32 unique protein-coding genes (PCGs). RSCU values over 1 indicate a significant codon use bias, whereas RSCU values below 1 imply a diminished codon usage bias in the protein-coding genes of the mitogenome (Figure 4, Table S5). The RSCU values of 29 codons in the protein-coding genes (PCGs) exceeded 1. Notably, the codon CGA (Arginine) in the atp9 gene exhibited the highest RSCU value of 6.0, reflecting extreme codon usage bias (Figure 4, Table S5). Furthermore, the RSCU values for nearly all codons containing an A/T in the third codon position are above 1.0. In contrast, the RSCU values for nearly all codons with a third codon position of C/G are  ≤  1.0 (Figure 4, Table S5). This occurrence suggests the significant prevalence of A/T in the third codon position in the mitogenome of *T. zeylanicus*, closely resembling findings in the mitogenomes of other terrestrial plants [53,54].

### 3.6. Analysis of Genes Under Selective Pressure

**Figure 5:**
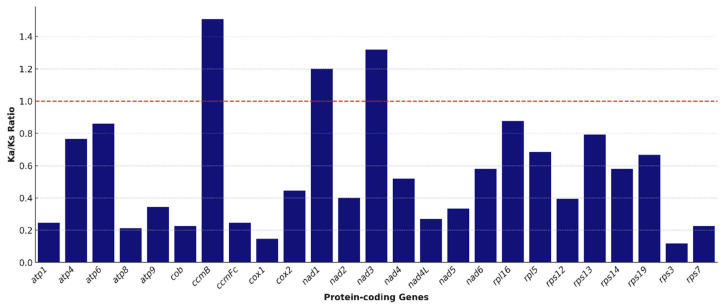
Genes under selection pressure. The Ka/Ks ratios of 24 mitochondrial protein-coding genes in *Trichopus zeylanicus*. Genes with Ka/Ks < 1 are considered to be under purifying selection, indicating functional conservation, while values > 1 suggest positive selection and potential adaptive evolution. The red dashed line represents the neutral selection threshold (Ka/Ks = 1). Ka—nonsynonymous substitution rate; Ks—synonymous substitution rate.

To investigate the evolutionary constraints acting on mitochondrial protein-coding genes (PCGs) of *T. zeylanicus*, the Ka/Ks (non-synonymous/synonymous substitution rate) ratios were calculated for 24 shared mitochondrial genes across four related monocot species: *A. officinalis*, *P. dactylifera*, *C. nucifera*, and *P. odorifer*. This analysis revealed that the majority of PCGs in *T. zeylanicus* are subject to strong purifying selection (Ka/Ks < 1), reflecting evolutionary pressure to maintain protein function and structural integrity in mitochondrial processes, particularly oxidative phosphorylation and ribosomal assembly [43,44]. Several genes showed low Ka/Ks values, indicative of evolutionary conservation. These include *cox1* (Ka/Ks = 0.16), *atp6* (Ka/Ks = 0.27), and *rps14* (Ka/Ks = 0.18) (Figure 5). *cox1* encodes a subunit of cytochrome c oxidase (Complex IV), a central component of the electron transport chain. Its high conservation is typical of plant mitochondria, where *cox1* is often used in phylogenetics due to its slow rate of evolution [55]. Similarly, atp6, a component of the ATP synthase complex, and rps14, a small-subunit ribosomal protein, also exhibit evolutionary stability, consistent with their essential roles in mitochondrial energy metabolism and translation, respectively. In contrast, three genes, *nad1* (Ka/Ks = 1.20), *nad3* (Ka/Ks = 1.49), and *ccmB* (Ka/Ks = 1.76), exhibited Ka/Ks ratios greater than 1.0, suggesting that they are evolving under positive selection (Figure 5). These genes may be undergoing adaptive evolution in response to selective pressures unique to *T. zeylanicus*, possibly linked to its ecological niche, altitude-specific respiration demands, or metabolic adaptations. *nad1* and *nad3* are part of Complex I (NADH dehydrogenase), and changes in these genes may affect mitochondrial respiration efficiency. Complex I is known for its structural plasticity and functional variability across plant lineages, particularly in stress-prone or energetically demanding environments [56]. ccmB, involved in cytochrome c maturation, showed the highest Ka/Ks value (1.76), pointing to significant evolutionary pressure. This suggests potential functional innovation or regulatory adaptation in cytochrome biogenesis, which is critical for efficient electron transport and reactive oxygen species management. These findings are consistent with broader trends observed in plant mitochondrial evolution, where the majority of PCGs are conserved but a subset show signs of accelerated evolution, often driven by environmental adaptation, subfunctionalization, or host–symbiont coevolution [57]. In *T. zeylanicus*, the presence of positively selected genes may reflect mitochondrial adaptations related to its medicinal properties, ecological specialization in the Western Ghats, or stress-resistance traits.

### 3.7. Chloroplast-Derived Mitochondrial Genome Sequences

**Figure 6:**
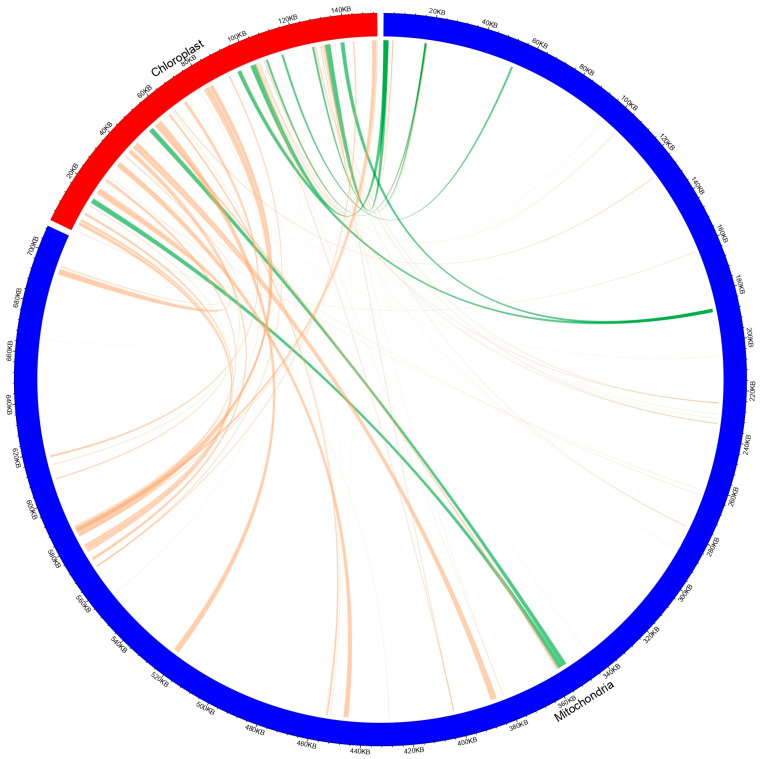
Chloroplast-to-mitochondrion sequence migration in the mitochondrial genome of *Trichopus zeylanicus*. The circular diagram shows regions of sequence homology between the chloroplast genome (red segment) and mitochondrial genome (blue segment). Orange lines indicate sequences in direct orientation, while green lines represent sequences in reverse orientation.

Genome exchange between mitochondria and chloroplasts is prevalent in plants. Approximately 5–10% of various species of the mitochondrial genome can identify homologous sequences in their corresponding chloroplast genome [58]. Homologous sections of the mitochondrial and chloroplast genomes of *T. zeylanicus* were identified by BlASTN (ncbi-blast-2.2.30+) analysis. Consequently, 80 pieces of 47,982 bp were detected migrating from the chloroplast to the mitochondrial genome of *T. zeylanicus*, representing 6.7% and 31.2% of the entire mitochondrial genome and chloroplast genome, respectively (Figure 6, Table S6). Additionally, nine annotated gene segments were transferred, comprising *rps12* (26%) and the complete sequences of eight tRNAs: *trnA-UGC*, *trnS-GGA*, *trnH-GUG*, *trnW-CCA,* *trnM-CAU*, *trnR-UCU*, *trnQ-UUG*, and *trnN-GUU* (Table S6). These observations suggest that several tRNA genes are amenable to transfer, exhibiting reduced sequence conservation during gene migration in *T. zeylanicus*.

### 3.8. Phylogenetic Analysis

**Figure 7:**
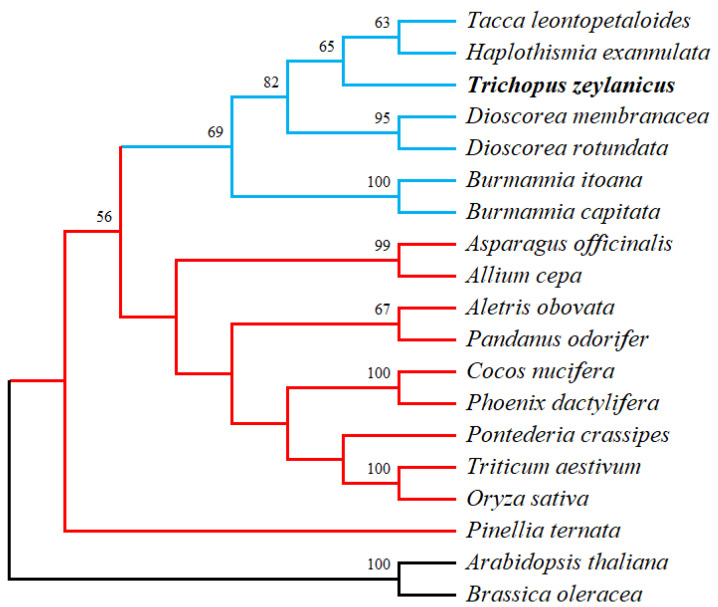
The phylogenetic analysis of *Trichopus zeylanicus* and closely related species based on six mitochondrial genes. The blue branches represent the species within the order Dioscoreales. The red branches denote other monocot species, illustrating the phylogenetic divergence between Dioscoreales and other monocot groups. The evolutionary history was inferred by using the Maximum Likelihood method and the Kimura 2-parameter model. The bootstrap values supporting the clustering are shown. Two dicots, Arabidopsis thaliana and Brassica oleracea, were used as outgroups.

The order Dioscoreales encompasses five families: Dioscoreaceae, Nartheciaceae, Taccaceae, Thismiaceae, and Burmanniaceae. *T. zeylanicus* is currently classified within the family Dioscoreaceae; however, its precise taxonomic position remains contentious [59]. To better understand its placement, we conducted a phylogenetic analysis using mitochondrial genome data. Given that a complete mitochondrial genome is unavailable for members of Dioscoreales (except for *T. zeylanicus*, which was sequenced in this study), we selected six mitochondrial genes—*atp1*, *ccmFc*, *cox2*, *mttB*, *nad4*, and *rps12*—for which sequences are available in most Dioscoreales members. Our analysis included three species from Burmanniaceae (*B. capitata*, *B. itoana*, and *Haplothismia exannulata*), two from Dioscoreaceae (*D. membranacea* and *D. cayenensis* subsp. *rotundata*), and one from Taccaceae (*T. leontopetaloides*), along with ten monocot species outside Dioscoreales (Figure 7, Table S7). Additionally, two dicot species (*Arabidopsis thaliana* and *Brassica oleracea*) were used as outgroups. Maximum likelihood (ML) phylogenetic analysis revealed that species within Dioscoreales (*D. membranacea*, *D. rotundata*, *T. zeylanicus*, *H. exannulata*, and *T. leontopetaloides*) formed a well-supported clade, distinct from other monocots. This finding reinforces the status of Dioscoreales as a distinct evolutionary lineage, highlighting its unique genetic and evolutionary characteristics among monocots (Figure 7). Within Dioscoreales, *T. zeylanicus* emerged as a separate subclade, showing a closer evolutionary relationship to *T. leontopetaloides*. This finding is consistent with our previous phylogenetic analyses based on both chloroplast and nuclear genomes, which also support the distinct placement and early divergence of *T. zeylanicus* within the order Dioscoreales. Notably, *H. exannulata* (Burmanniaceae) clustered with *T. leontopetaloides* (Taccaceae) rather than grouping with other Dioscoreaceae members, underscoring potential complexities in the evolutionary relationships within the order. Interestingly, this pattern aligns with the nuclear 18S rDNA phylogeny by Merckx et al. (2006), which placed the tribe Thismieae (including Haplothismia) as a sister to Taccaceae [60]. This suggests that the observed clustering may reflect a true evolutionary relationship and highlights the need for multilocus data to resolve deep nodes within Dioscoreales.

## 4. Conclusions

This study presents the first complete mitochondrial genome assembly and comprehensive analysis for *T. zeylanicus*, shedding light on its structural organization, gene content, repetitive elements, RNA editing, and interorganellar gene transfer. The mitogenome of *T. zeylanicus* demonstrates notable features, including moderate RNA editing, chloroplast-derived sequences, and a unique repertoire of repetitive elements, highlighting its evolutionary adaptation and complex mitogenomic architecture. Our phylogenetic analysis highlights its distinct placement within the order Dioscoreales, reinforcing its unique genetic lineage. As the first mitogenome characterized within this order, this research provides a valuable resource for advancing our understanding of mitochondrial genome evolution, functional genomics, and phylogenetics in angiosperms, particularly in Dioscoreales and related lineages.

## Figures and Tables

**Table 3:** Details of tandem repeats in the mitochondrial genome of *T. zeylanicus*.

No.	Size	Copy	Start	End	Sequence
1	25	3	35,388	35,462	GTCTCATAGGTTACATGGAATACCG
2	17	2	127,171	127,204	AGTGAATCAGATCGTAG
3	27	2	127,199	127,252	TGGTAGTTCGCGTGCTCAAGTGAAATG
4	17	2	141,404	141,440	CAAGGCAAGGTCAGGCT
5	17	3	141,404	141,446	CAAGGCAAGGTCAGGCT
6	17	2	201,046	201,079	AACCCTGATCGTCTTCC
7	22	3	219,866	219,933	CGTGCATGTCACCGTCTCCACC
8	18	2	224,271	224,308	TGTTGTTGCAATACCCGT
9	13	2	254,962	254,986	TCAAAGTGAGAAC
10	13	2	381,379	381,403	TTGTATATCCAAA
11	19	2	385,131	385,169	AATAGTAATAGTTCTATTC
12	13	2	458,381	458,411	TAGCCTCTAACTC
13	25	2	472,717	472,764	GTAGCATGAAGAAAGCAGAAGTGGA
14	27	2	487,362	487,427	ATACTTGCAGCGGGGATTCTACCTCTT
15	21	2	536,618	536,665	TCGGCGTCCGTCTATCTATTG
16	21	2	570,837	570,876	TTGATAATCCTACTCTTTTCC
17	25	3	596,343	596,417	CGAAGAAAGCACTACACCTGGCAGG
18	14	2	691,088	691,117	AGGGACTGCCTGGAA

## Data Availability

The datasets analyzed in this study can be found in online repositories. The names of the repositories and accession numbers can be found in the article (Table 2 and Table S7). Additional data generated in this study can be found in the Supplementary Materials.

## Supplementary Materials

The following supporting information can be downloaded at: https://www.mdpi.com/article/10.3390/cimb47070553/s1.
