# Supplementary material for: Assembly and Comparative Analysis of Complete Mitochondrial Genome Sequence of Endangered Medicinal Plant Trichopus zeylanicus
Source: Curr Issues Mol Biol. 2025 Jul 16;47(7):553. doi: 10.3390/cimb47070553 (PMC12293161; doi:10.3390/cimb47070553)
Supplement: Supplementary file 1 [file cimb-47-00553-s001.zip › Figure S1 and S2.pdf]

Assembly and comparative analysis of complete mitochondrial genome sequence of an endangered medicinal plant *Trichopus zeylanicus*

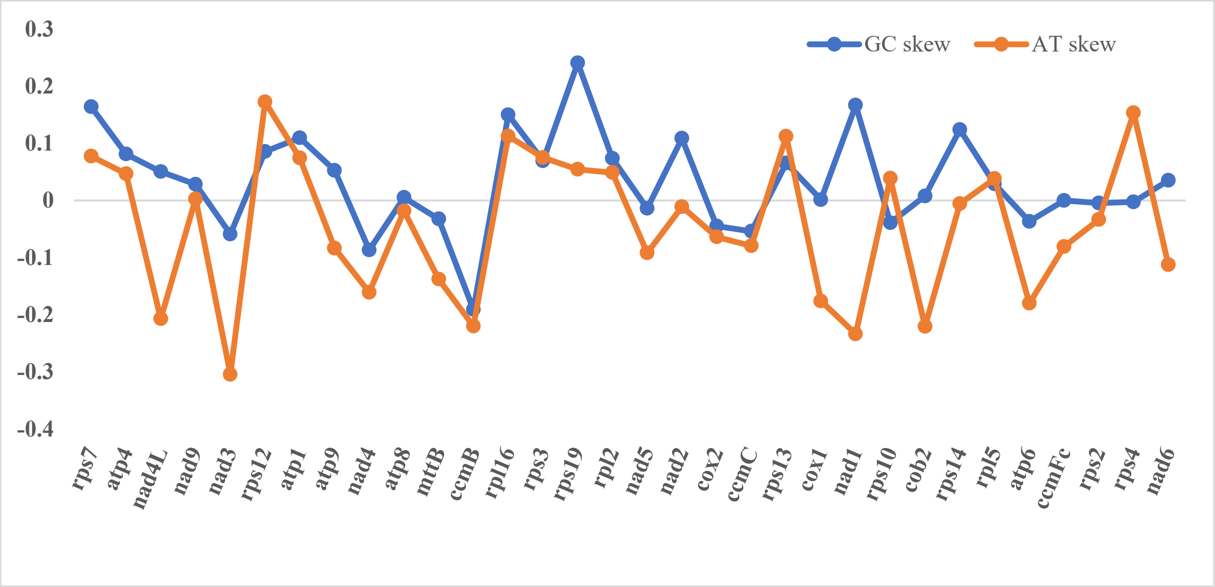

Figure S1. AT and GC skews in 29 protein coding genes of the mitochondrial genome of *Trichopus zeylanicus*. AT skew, determined as (A - T) / (A + T), and GC skew, determined as (G - C) / (G + C).

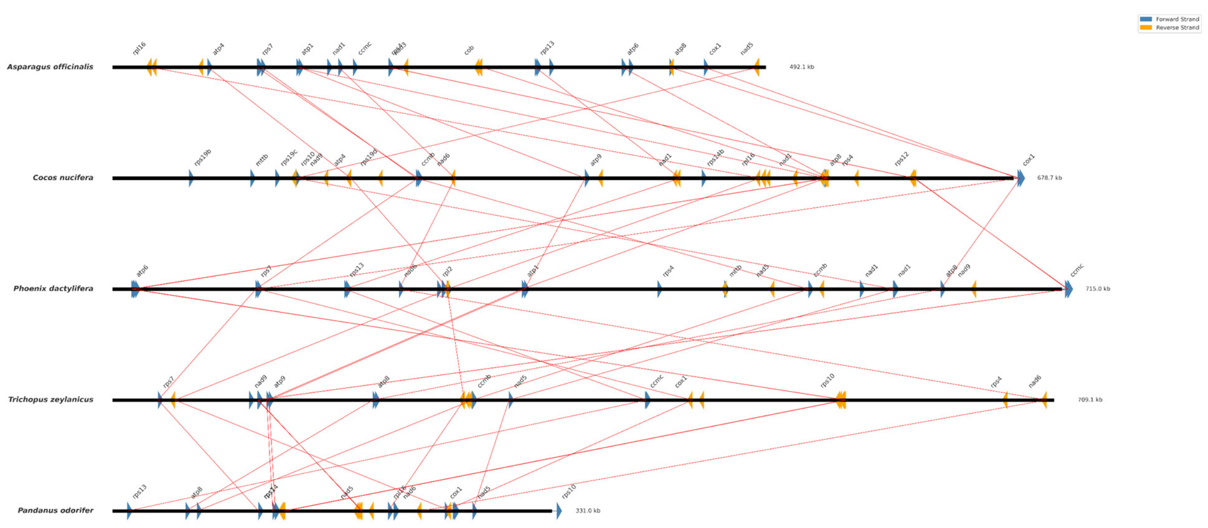

Figure S2. Comparative synteny map of mitochondrial genomes from five monocot species: Asparagus officinalis, Cocos nucifera, Phoenix dactylifera, Trichopus zeylanicus, and Pandanus odorifer. Conserved gene blocks and homologous regions are connected by red lines, illustrating shared syntenic relationships. Gene orientation is indicated by arrow direction, with forward strand genes in blue and reverse strand genes in orange
