# Supplementary material for: Assembly and Comparative Analysis of Complete Mitochondrial Genome Sequence of Endangered Medicinal Plant Trichopus zeylanicus
Source: Curr Issues Mol Biol. 2025 Jul 16;47(7):553. doi: 10.3390/cimb47070553 (PMC12293161; doi:10.3390/cimb47070553)
Supplement: Supplementary file 1 [file cimb-47-00553-s001.zip › Supplementary Tables.pdf]

Assembly and comparative analysis of complete mitochondrial genome sequence of an endangered medicinal plant *Trichopus zeylanicus*

Supplementary Tables S1, S2, S3, S4 and S6 and S7

Table S1. Mitochondrial genome features of Trichopus zeylanicus

**Table S1. Mitochondrial genome features of *Trichopus zeylanicus***					
Genome	Feature	Start	End	Strand	Id
Trichopus	gene	417	1224	-	ID=trnA-UGC;Name=trnA-UGC
Trichopus	gene_component_region	1120	1224	-	Parent=trnA-UGC;Name=trnA-UGC
Trichopus	tRNA	417	1224	-	ID=trnA-UGC.r01;Parent=trnA-UGC;Name=trnA-UGC
Trichopus	tRNA	1120	1224	-	ID=trnA-UGC.r02;Parent=trnA-UGC;Name=trnA-UGC
Trichopus	exon	417	452	-	Parent=trnA-UGC.r01;Name=trnA-UGC
Trichopus	exon	1188	1224	-	Parent=trnA-UGC.r01;Name=trnA-UGC
Trichopus	exon	1120	1224	-	Parent=trnA-UGC.r02;Name=trnA-UGC
Trichopus	gene	22354	22425	-	ID=trnC-GCA;Name=trnC-GCA
Trichopus	tRNA	22354	22425	-	ID=trnC-GCA.r01;Parent=trnC-GCA;Name=trnC-GCA
Trichopus	tRNA	22355	22425	-	ID=trnC-GCA.r02;Parent=trnC-GCA;Name=trnC-GCA
Trichopus	exon	22354	22425	-	Parent=trnC-GCA.r01;Name=trnC-GCA
Trichopus	exon	22355	22425	-	Parent=trnC-GCA.r02;Name=trnC-GCA
Trichopus	gene	29027	29473	+	ID=rps7;Name=rps7
Trichopus	mRNA	29027	29473	+	ID=rps7.t01;Parent=rps7
Trichopus	CDS	29027	29473	+	ID=rps7.p01;Parent=rps7.t01;Name=rps7;codon_start=1;product=ribosomal protein S7,RPS7;translation=length.148
Trichopus	exon	29027	29473	+	Parent=rps7.t01
Trichopus	gene	30781	30889	+	ID=trnS-UGA;Name=trnS-UGA
Trichopus	tRNA	30781	30889	+	ID=trnS-UGA.r01;Parent=trnS-UGA;Name=trnS-UGA

Trichopus	exon	30781	30810	+	Parent=trnS-UGA.r01;Name=trnS-UGA
Trichopus	exon	30830	30889	+	Parent=trnS-UGA.r01;Name=trnS-UGA
Trichopus	gene	51309	51896	-	ID=atp4;Name=atp4
Trichopus	mRNA	51309	51896	-	ID=atp4.t01;Parent=atp4
Trichopus	CDS	51309	51896	-	ID=atp4.p01;Parent=atp4.t01;Name=atp4;codon_start=1;product=ATP synthase subunit 4,ATP4;translation=length.195
Trichopus	exon	51309	51896	-	Parent=atp4.t01
Trichopus	gene	52043	52315	-	ID=nad4L;Name=nad4L
Trichopus	mRNA	52043	52315	-	ID=nad4L.t01;Parent=nad4L
Trichopus	CDS	52043	52315	-	ID=nad4L.p01;Parent=nad4L.t01;Name=nad4L;codon_start=1;product=NADH dehydrogenase subunit 4L,NADH4L;translation=length.90
Trichopus	exon	52043	52315	-	Parent=nad4L.t01
Trichopus	gene	556125	559314	+	ID=ccmFc;Name=ccmFc
Trichopus	mRNA	556125	559314	+	ID=ccmFc.t01;Parent=ccmFc
Trichopus	CDS	556125	556946	+	ID=ccmFc.p01;Parent=ccmFc.t01;Name=ccmFc;codon_start=1;product=Cytochrome c biogenesis CcmF C-terminal-like mitochondrial protein,CCMFC;translation=length.635
Trichopus	CDS	557069	557123	+	ID=ccmFc.p01;Parent=ccmFc.t01;Name=ccmFc;codon_start=1;product=Cytochrome c biogenesis CcmF C-terminal-like mitochondrial protein,CCMFC;translation=length.635
Trichopus	CDS	557287	557435	+	ID=ccmFc.p01;Parent=ccmFc.t01;Name=ccmFc;codon_start=1;product=Cytochrome c biogenesis CcmF C-terminal-like mitochondrial protein,CCMFC;translation=length.635
Trichopus	CDS	557547	558247	+	ID=ccmFc.p01;Parent=ccmFc.t01;Name=ccmFc;codon_start=1;product=Cytochrome c biogenesis CcmF C-terminal-like mitochondrial protein,CCMFC;translation=length.635
Trichopus	CDS	558405	558524	+	ID=ccmFc.p01;Parent=ccmFc.t01;Name=ccmFc;codon_start=1;product=Cytochrome c biogenesis CcmF C-terminal-like mitochondrial protein,CCMFC;translation=length.635
Trichopus	CDS	559254	559314	+	ID=ccmFc.p01;Parent=ccmFc.t01;Name=ccmFc;codon_start=1;product=Cytochrome c biogenesis CcmF C-terminal-like mitochondrial protein,CCMFC;translation=length.635
Trichopus	exon	556125	556946	+	Parent=ccmFc.t01;Name=ccmFc;number=1

Trichopus	exon	557069	557123	+	Parent=ccmFc.t01;Name=ccmFc;number=2
Trichopus	exon	557287	557435	+	Parent=ccmFc.t01;Name=ccmFc;number=3
Trichopus	exon	557547	558247	+	Parent=ccmFc.t01;Name=ccmFc;number=4
Trichopus	exon	558405	558524	+	Parent=ccmFc.t01
Trichopus	exon	559254	559314	+	Parent=ccmFc.t01;Name=ccmFc;number=6
Trichopus	gene	96894	97469	+	ID=nad9;Name=nad9
Trichopus	mRNA	96894	97469	+	ID=nad9.t01;Parent=nad9
Trichopus	CDS	96894	97469	+	ID=nad9.p01;Parent=nad9.t01;Name=nad9;codon_start=1;product=NADH dehydrogenase subunit 9,NADH9;translation=length.191
Trichopus	exon	96894	97469	+	Parent=nad9.t01
Trichopus	gene	98086	98169	+	ID=trnY-GUA;Name=trnY-GUA
Trichopus	tRNA	98086	98169	+	ID=trnY-GUA.r01;Parent=trnY-GUA;Name=trnY-GUA
Trichopus	exon	98086	98169	+	Parent=trnY-GUA.r01;Name=trnY-GUA
Trichopus	gene	98086	98168	+	ID=trnY-GUA | trnY;Name=trnY-GUA | trnY
Trichopus	tRNA	98086	98168	+	ID=trnY-GUA | trnY.r01;Parent=trnY-GUA | trnY;Name=trnY-GUA | trnY
Trichopus	exon	98086	98168	+	Parent=trnY-GUA | trnY.r01;Name=trnY-GUA | trnY
Trichopus	gene	103054	103410	+	ID=nad3;Name=nad3
Trichopus	mRNA	103054	103410	+	ID=nad3.t01;Parent=nad3
Trichopus	CDS	103054	103410	+	ID=nad3.p01;Parent=nad3.t01;Name=nad3;codon_start=1;product=NADH dehydrogenase subunit 3,NADH3;translation=length.118
Trichopus	exon	103054	103410	+	Parent=nad3.t01
Trichopus	gene	103455	103832	+	ID=rps12;Name=rps12
Trichopus	mRNA	103455	103832	+	ID=rps12.t01;Parent=rps12
Trichopus	CDS	103455	103832	+	ID=rps12.p01;Parent=rps12.t01;Name=rps12;codon_start=1;product=Ribosomal protein S12,RPS12;translation=length.125
Trichopus	exon	103455	103832	+	Parent=rps12.t01
Trichopus	tRNA	106669	106753	-	ID=trnQ-UUA;Name=trnQ-UUA
Trichopus	exon	106669	106703	-	ID=trnQ-UUA.exon;Alias=trnQ-UUA;Name=trnQ-UUA
Trichopus	exon	106716	106753	-	ID=trnQ-UUA.exon.1;Alias=trnQ-UUA;Name=trnQ-UUA
Trichopus	tRNA	106669	106753	-	ID=trnTERM-UUA;Name=trnTERM-UUA
Trichopus	exon	106669	106753	-	ID=trnTERM-UUA.exon;Alias=trnTERM-UUA;Name=trnTERM-UUA

Trichopus	gene	106669	106753	-		ID=trnTERM-UUA | trnQ-UUA;Name=trnTERM-UUA | trnQ-UUA
Trichopus	gene	109715	111268	+		ID=atp1;Name=atp1
Trichopus	mRNA	109715	111268	+		ID=atp1.t01;Parent=atp1
Trichopus	CDS	109715	111268	+		ID=atp1.p01;Parent=atp1.t01;Name=atp1;codon_start=1;product=ATP synthase subunit 1,ATP1;translation=length.517
Trichopus	exon	109715	111268	+		Parent=atp1.t01
Trichopus	gene	111451	111708	+		ID=atp9;Name=atp9
Trichopus	mRNA	111451	111708	+		ID=atp9.t01;Parent=atp9
Trichopus	CDS	111451	111708	+		ID=atp9.p01;Parent=atp9.t01;Name=atp9;codon_start=1;product=ATP synthase subunit 9,ATP9;translation=length.85
Trichopus	exon	111451	111708	+		Parent=atp9.t01
Trichopus	gene	128446	137356	-		ID=nad4;Name=nad4
Trichopus	mRNA	128446	137356	-		ID=nad4.t01;Parent=nad4
Trichopus	CDS	128446	128535	-		ID=nad4.p01;Parent=nad4.t01;Name=nad4;codon_start=1;product=NAD H dehydrogenase 4,NADH4;translation=length.495
Trichopus	CDS	130608	131030	-		ID=nad4.p01;Parent=nad4.t01;Name=nad4;codon_start=1;product=NAD H dehydrogenase 4,NADH4;translation=length.495
Trichopus	CDS	135019	135531	-		ID=nad4.p01;Parent=nad4.t01;Name=nad4;codon_start=1;product=NAD H dehydrogenase 4,NADH4;translation=length.495
Trichopus	CDS	136895	137356	-		ID=nad4.p01;Parent=nad4.t01;Name=nad4;codon_start=1;product=NAD H dehydrogenase 4,NADH4;translation=length.495
Trichopus	exon	128446	128535	-		Parent=nad4.t01;Name=nad4;number=4
Trichopus	exon	130608	131030	-		Parent=nad4.t01;Name=nad4;number=3
Trichopus	exon	135019	135531	-		Parent=nad4.t01;Name=nad4;number=2
Trichopus	exon	136895	137356	-		Parent=nad4.t01;Name=nad4;number=1
Trichopus	gene	159070	159159	+		ID=trnS-GCU;Name=trnS-GCU
Trichopus	tRNA	159070	159159	+		ID=trnS-GCU.r01;Parent=trnS-GCU;Name=trnS-GCU
Trichopus	exon	159070	159159	+		Parent=trnS-GCU.r01;Name=trnS-GCU
Trichopus	gene	188689	189150	+		ID=atp8;Name=atp8
Trichopus	mRNA	188689	189150	+		ID=atp8.t01;Parent=atp8
Trichopus	CDS	188689	189150	+		ID=atp8.p01;Parent=atp8.t01;Name=atp8;codon_start=1;product=ATP synthase subunit 8,ATP8;translation=length.153

Trichopus	exon	188689	189150	+	Parent=atp8.t01
Trichopus	gene	190462	191020	+	ID=mttB;Name=mttB
Trichopus	mRNA	190462	191020	+	ID=mttB.t01;Parent=mttB
Trichopus	CDS	190462	190680	+	ID=mttB.p01;Parent=mttB.t01;Name=mttB;codon_start=1;product=Trimethylamine methyltransferase,MTTB;translation=length.161
Trichopus	CDS	190754	191020	+	ID=mttB.p01;Parent=mttB.t01;Name=mttB;codon_start=1;product=Trimethylamine methyltransferase,MTTB;translation=length.161
Trichopus	exon	190462	190680	+	Parent=mttB.t01;Name=mttB;number=1
Trichopus	exon	190754	191020	+	Parent=mttB.t01;Name=mttB;number=2
Trichopus	gene	206256	206328	+	ID=trnM-CAU;Name=trnM-CAU
Trichopus	tRNA	206256	206328	+	ID=trnM-CAU.r01;Parent=trnM-CAU;Name=trnM-CAU
Trichopus	exon	206256	206328	+	Parent=trnM-CAU.r01;Name=trnM-CAU
Trichopus	gene	240225	240298	-	ID=trnM-CAU.gene;Alias=trnM-CAU;Name=trnM-CAU
Trichopus	tRNA	240225	240298	-	ID=trnM-CAU.r01.tRNA;Parent=trnM-CAU;Alias=trnM-CAU.r01;Name=trnM-CAU
Trichopus	exon	240225	240298	-	Parent=trnM-CAU.r01;Name=trnM-CAU
Trichopus	gene	229879	230861	+	ID=18Srrn | rrn18;Name=18Srrn | rrn18
Trichopus	rRNA	229879	230861	+	ID=18Srrn | rrn18.r01;Parent=18Srrn | rrn18;Name=18Srrn | rrn18
Trichopus	exon	229879	230861	+	Parent=18Srrn | rrn18.r01;Name=18Srrn | rrn18
Trichopus	gene	231712	231825	+	ID=5Srrn;Name=5Srrn
Trichopus	rRNA	231712	231825	+	ID=5Srrn.r01;Parent=5Srrn;Name=5Srrn
Trichopus	exon	231712	231825	+	Parent=5Srrn.r01;Name=5Srrn
Trichopus	gene	231713	231825	+	ID=rrn5;Name=rrn5
Trichopus	rRNA	231713	231825	+	ID=rrn5.r01;Parent=rrn5;Name=rrn5
Trichopus	exon	231713	231825	+	Parent=rrn5.r01;Name=rrn5
Trichopus	gene	233712	233752	-	ID=trnN-GUU;Name=trnN-GUU
Trichopus	tRNA	233712	233752	-	ID=trnN-GUU.r01;Parent=trnN-GUU;Name=trnN-GUU
Trichopus	exon	233712	233752	-	Parent=trnN-GUU.r01;Name=trnN-GUU
Trichopus	region	242970	243043	+	ID=psbA;Name=psbA
Trichopus	gene	342989	343029	+	ID=trnN-GUU.gene;Alias=trnN-GUU;Name=trnN-GUU

Trichopus	tRNA	342989	343029	+	ID=trnN-GUU.r01.tRNA;Parent=trnN-GUU;Alias=trnN-GUU.r01;Name=trnN-GUU
Trichopus	exon	342989	343029	+	Parent=trnN-GUU.r01;Name=trnN-GUU
Trichopus	gene	246335	246394	-	ID=26Srrn | rrn26;Name=26Srrn | rrn26
Trichopus	rRNA	246335	246394	-	ID=26Srrn | rrn26.r01;Parent=26Srrn | rrn26;Name=26Srrn | rrn26
Trichopus	exon	246335	246394	-	Parent=26Srrn | rrn26.r01;Name=26Srrn | rrn26
Trichopus	gene	262790	263410	+	ID=ccmB;Name=ccmB
Trichopus	mRNA	262790	263410	+	ID=ccmB.t01;Parent=ccmB
Trichopus	CDS	262790	263410	+	ID=ccmB.p01;Parent=ccmB.t01;Name=ccmB;codon_start=1;product=cytochrome c biogenesis protein,CCMB;translation=length.206
Trichopus	exon	262790	263410	+	Parent=ccmB.t01
Trichopus	gene	266401	266958	-	ID=rpl16;Name=rpl16
Trichopus	mRNA	266401	266958	-	ID=rpl16.t01;Parent=rpl16
Trichopus	CDS	266401	266958	-	ID=rpl16.p01;Parent=rpl16.t01;Name=rpl16;codon_start=1;product=Large ribosomal subunit protein uL16,RPL16;translation=length.185
Trichopus	exon	266401	266958	-	Parent=rpl16.t01
Trichopus	gene	266807	268447	-	ID=rps3;Name=rps3
Trichopus	mRNA	266807	268447	-	ID=rps3.t01;Parent=rps3
Trichopus	CDS	266807	268447	-	ID=rps3.p01;Parent=rps3.t01;Name=rps3;codon_start=1;product=Small ribosomal subunit protein eS3,RPS3;translation=length.546
Trichopus	exon	266807	268447	-	Parent=rps3.t01
Trichopus	gene	270254	270526	-	ID=rps19;Name=rps19
Trichopus	mRNA	270254	270526	-	ID=rps19.t01;Parent=rps19
Trichopus	CDS	270254	270526	-	ID=rps19.p01;Parent=rps19.t01;Name=rps19;codon_start=1;product=Small ribosomal subunit protein eS19,RPS19;translation=length.90
Trichopus	exon	270254	270526	-	Parent=rps19.t01
Trichopus	gene	272080	273528	-	ID=rpl2;Name=rpl2
Trichopus	mRNA	272080	273528	-	ID=rpl2.t01;Parent=rpl2
Trichopus	CDS	272080	272382	-	ID=rpl2.p01;Parent=rpl2.t01;Name=rpl2;codon_start=1;product=Large ribosomal subunit protein uL2,RPL2;translation=length.387
Trichopus	CDS	272668	273528	-	ID=rpl2.p01;Parent=rpl2.t01;Name=rpl2;codon_start=1;product=Large ribosomal subunit protein uL2,RPL2;translation=length.387

Trichopus	exon	272080	272382	-	Parent=rpl2.t01;Name=rpl2;number=2
Trichopus	exon	272668	273528	-	Parent=rpl2.t01;Name=rpl2;number=1
Trichopus	gene	289924	292627	+	ID=nad5;Name=nad5
Trichopus	mRNA	289924	292627	+	ID=nad5.t01;Parent=nad5
Trichopus	CDS	289924	290134	+	ID=nad5.p01;Parent=nad5.t01;Name=nad5;codon_start=1;product=NADH dehydrogenase 5,NADH5;translation=length.664
Trichopus	CDS	290584	291977	+	ID=nad5.p01;Parent=nad5.t01;Name=nad5;codon_start=1;product=NADH dehydrogenase 5,NADH5;translation=length.664
Trichopus	CDS	292238	292627	+	ID=nad5.p01;Parent=nad5.t01;Name=nad5;codon_start=1;product=NADH dehydrogenase 5,NADH5;translation=length.664
Trichopus	exon	289924	290134	+	Parent=nad5.t01;Name=nad5;number=1
Trichopus	exon	290584	291977	+	Parent=nad5.t01;Name=nad5;number=2
Trichopus	exon	292238	292627	+	Parent=nad5.t01;Name=nad5;number=3
Trichopus	gene	321806	322971	-	ID=nad2;Name=nad2
Trichopus	mRNA	321806	322971	-	ID=nad2.t01;Parent=nad2
Trichopus	CDS	321806	321994	-	ID=nad2.p01;Parent=nad2.t01;Name=nad2;codon_start=1;product=NADH dehydrogenase 2,NADH2;translation=length.251
Trichopus	CDS	322262	322461	-	ID=nad2.p01;Parent=nad2.t01;Name=nad2;codon_start=1;product=NADH dehydrogenase 2,NADH2;translation=length.251
Trichopus	CDS	322605	322971	-	ID=nad2.p01;Parent=nad2.t01;Name=nad2;codon_start=1;product=NADH dehydrogenase 2,NADH2;translation=length.251
Trichopus	exon	321806	321994	-	Parent=nad2.t01;Name=nad2;number=3
Trichopus	exon	322262	322461	-	Parent=nad2.t01;Name=nad2;number=2
Trichopus	exon	322605	322971	-	Parent=nad2.t01;Name=nad2;number=1
Trichopus	gene	375260	378846	+	ID=cox2;Name=cox2
Trichopus	mRNA	375260	378846	+	ID=cox2.t01;Parent=cox2
Trichopus	CDS	375260	375639	+	ID=cox2.p01;Parent=cox2.t01;Name=cox2;codon_start=1;product=Cytochrome c oxidase subunit 2,COX2;translation=length.256
Trichopus	CDS	376882	377197	+	ID=cox2.p01;Parent=cox2.t01;Name=cox2;codon_start=1;product=Cytochrome c oxidase subunit 2,COX2;translation=length.256
Trichopus	CDS	378772	378846	+	ID=cox2.p01;Parent=cox2.t01;Name=cox2;codon_start=1;product=Cytochrome c oxidase subunit 2,COX2;translation=length.256

Trichopus	exon	375260	375639	+	Parent=cox2.t01;Name=cox2;number=1
Trichopus	exon	376882	377197	+	Parent=cox2.t01;Name=cox2;number=2
Trichopus	exon	378772	378846	+	Parent=cox2.t01;Name=cox2;number=3
Trichopus	gene	384902	384988	-	ID=trnS-GGA | trnS;Name=trnS-GGA | trnS
Trichopus	tRNA	384902	384988	-	ID=trnS-GGA | trnS.r01;Parent=trnS-GGA | trnS;Name=trnS-GGA | trnS
Trichopus	exon	384902	384988	-	Parent=trnS-GGA | trnS.r01;Name=trnS-GGA | trnS
Trichopus	gene	391130	392050	+	ID=ccmC;Name=ccmC
Trichopus	mRNA	391130	392050	+	ID=ccmC.t01;Parent=ccmC
Trichopus	CDS	391130	392050	+	ID=ccmC.p01;Parent=ccmC.t01;Name=ccmC;codon_start=1;product=cytochrome c maturation C protein,CCMC;translation=length.306
Trichopus	exon	391130	392050	+	Parent=ccmC.t01
Trichopus	gene	391939	392247	+	ID=rps13;Name=rps13
Trichopus	mRNA	391939	392247	+	ID=rps13.t01;Parent=rps13
Trichopus	CDS	391939	392247	+	ID=rps13.p01;Parent=rps13.t01;Name=rps13;codon_start=1;product=Small ribosomal subunit protein eS19,RPS13;translation=length.102
Trichopus	exon	391939	392247	+	Parent=rps13.t01
Trichopus	gene	435945	437528	-	ID=cox1;Name=cox1
Trichopus	mRNA	435945	437528	-	ID=cox1.t01;Parent=cox1
Trichopus	CDS	435945	437528	-	ID=cox1.p01;Parent=cox1.t01;Name=cox1;codon_start=1;product=Cytochrome c oxidase subunit 1,COX1;translation=length.527
Trichopus	exon	435945	437528	-	Parent=cox1.t01
Trichopus	gene	444412	444711	-	ID=nad1;Name=nad1
Trichopus	mRNA	444412	444711	-	ID=nad1.t01;Parent=nad1
Trichopus	CDS	444412	444711	-	ID=nad1.p01;Parent=nad1.t01;Name=nad1;codon_start=1;product=NADH dehydrogenase subunit 1,NAD1;translation=length.99
Trichopus	exon	444412	444711	-	Parent=nad1.t01
Trichopus	gene	453213	453286	+	ID=trnW-CCA;Name=trnW-CCA
Trichopus	tRNA	453213	453286	+	ID=trnW-CCA.r01;Parent=trnW-CCA;Name=trnW-CCA
Trichopus	exon	453213	453286	+	Parent=trnW-CCA.r01;Name=trnW-CCA
Trichopus	gene	545578	545937	-	ID=rps10;Name=rps10
Trichopus	mRNA	545578	545937	-	ID=rps10.t01;Parent=rps10

Trichopus	CDS	545578	545937	-	ID=rps10.p01;Parent=rps10.t01;Name=rps10;codon_start=1;product=Small ribosomal subunit protein eS10,RPS10;translation=length.119
Trichopus	exon	545578	545937	-	Parent=rps10.t01
Trichopus	gene	546973	548151	-	ID=cob2 | cob;Name=cob2 | cob
Trichopus	mRNA	546973	548151	-	ID=cob2 | cob.t01;Parent=cob2 | cob
Trichopus	CDS	546973	548151	-	ID=cob2 | cob.p01;Parent=cob2 | cob.t01;Name=cob2 | cob;codon_start=1;product=Cytochrome b\,COB2;translation=length.392
Trichopus	exon	546973	548151	-	Parent=cob2 | cob.t01
Trichopus	gene	548985	549302	-	ID=rps14;Name=rps14
Trichopus	mRNA	548985	549302	-	ID=rps14.t01;Parent=rps14
Trichopus	CDS	548985	549302	-	ID=rps14.p01;Parent=rps14.t01;Name=rps14;codon_start=1;product=Small ribosomal subunit protein uS14,RPS14;translation=length.105
Trichopus	exon	548985	549302	-	Parent=rps14.t01
Trichopus	gene	549289	549843	-	ID=rpl5;Name=rpl5
Trichopus	mRNA	549289	549843	-	ID=rpl5.t01;Parent=rpl5
Trichopus	CDS	549289	549843	-	ID=rpl5.p01;Parent=rpl5.t01;Name=rpl5;codon_start=1;product=Large ribosomal subunit protein uL15,RPL15;translation=length.184
Trichopus	exon	549289	549843	-	Parent=rpl5.t01
Trichopus	gene	550010	550876	-	ID=atp6;Name=atp6
Trichopus	mRNA	550010	550876	-	ID=atp6.t01;Parent=atp6
Trichopus	CDS	550010	550876	-	ID=atp6.p01;Parent=atp6.t01;Name=atp6;codon_start=1;product=ATP synthase subunit 6,ATP6;translation=length.288
Trichopus	exon	550010	550876	-	Parent=atp6.t01
Trichopus	gene	576175	576247	-	ID=trnR-UCU;Name=trnR-UCU
Trichopus	tRNA	576175	576247	-	ID=trnR-UCU.r01;Parent=trnR-UCU;Name=trnR-UCU
Trichopus	tRNA	576182	576246	-	ID=trnR-UCU.r02;Parent=trnR-UCU;Name=trnR-UCU
Trichopus	exon	576175	576247	-	Parent=trnR-UCU.r01;Name=trnR-UCU
Trichopus	exon	576182	576246	-	Parent=trnR-UCU.r02;Name=trnR-UCU
Trichopus	gene	578891	578962	-	ID=trnQ-UUG;Name=trnQ-UUG
Trichopus	tRNA	578891	578962	-	ID=trnQ-UUG.r01;Parent=trnQ-UUG;Name=trnQ-UUG
Trichopus	exon	578891	578962	-	Parent=trnQ-UUG.r01;Name=trnQ-UUG
Trichopus	gene	579263	579853	-	ID=rps2;Name=rps2

Trichopus	mRNA	579263	579853	-	ID=rps2.t01;Parent=rps2
Trichopus	CDS	579263	579853	-	ID=rps2.p01;Parent=rps2.t01;Name=rps2;codon_start=1;product=Small ribosomal subunit protein uS2,RPS2;translation=length.196
Trichopus	exon	579263	579853	-	Parent=rps2.t01
Trichopus	gene	583071	583145	+	ID=trnH-GUG;Name=trnH-GUG
Trichopus	tRNA	583071	583145	+	ID=trnH-GUG.r01;Parent=trnH-GUG;Name=trnH-GUG
Trichopus	exon	583071	583145	+	Parent=trnH-GUG.r01;Name=trnH-GUG
Trichopus	gene	670068	671078	-	ID=rps4;Name=rps4
Trichopus	mRNA	670068	671078	-	ID=rps4.t01;Parent=rps4
Trichopus	CDS	670068	671078	-	ID=rps4.p01;Parent=rps4.t01;Name=rps4;codon_start=1;product=Small ribosomal subunit protein uS4,RPS4;translation=length.336
Trichopus	exon	670068	671078	-	Parent=rps4.t01
Trichopus	gene	699286	700080	-	ID=nad6;Name=nad6
Trichopus	mRNA	699286	700080	-	ID=nad6.t01;Parent=nad6
Trichopus	CDS	699286	700080	-	ID=nad6.p01;Parent=nad6.t01;Name=nad6;codon_start=1;product=NADH dehydrogenase 6,NADH6;translation=length.264
Trichopus	exon	699286	700080	-	Parent=nad6.t01
# GFF3 saved to stdout25889					

Table S2. Simple Sequence repeats in the mitochondrial genome of *Trichopus zeylanicus*

Si. No	genome	start	end	repeat	Type
1	Mitochondria	22752	22761	(A)10	monomeric_repeat
2	Mitochondria	31120	31129	(T)10	monomeric_repeat
3	Mitochondria	89023	89032	(A)10	monomeric_repeat
4	Mitochondria	96634	96643	(A)10	monomeric_repeat
5	Mitochondria	134844	134853	(T)10	monomeric_repeat
6	Mitochondria	141476	141486	(A)11	monomeric_repeat
7	Mitochondria	164043	164052	(A)10	monomeric_repeat
8	Mitochondria	377725	377734	(A)10	monomeric_repeat
9	Mitochondria	396658	396667	(A)10	monomeric_repeat
10	Mitochondria	437741	437750	(A)10	monomeric_repeat
11	Mitochondria	453605	453614	(T)10	monomeric_repeat
12	Mitochondria	468164	468173	(A)10	monomeric_repeat
13	Mitochondria	520319	520328	(A)10	monomeric_repeat
14	Mitochondria	556488	556497	(T)10	monomeric_repeat
15	Mitochondria	576163	576172	(A)10	monomeric_repeat
16	Mitochondria	582368	582379	(G)12	monomeric_repeat
17	Mitochondria	603372	603381	(A)10	monomeric_repeat
18	Mitochondria	611223	611232	(T)10	monomeric_repeat
19	Mitochondria	630438	630447	(A)10	monomeric_repeat
20	Mitochondria	636651	636660	(T)10	monomeric_repeat
21	Mitochondria	640012	640021	(T)10	monomeric_repeat
22	Mitochondria	670060	670069	(T)10	monomeric_repeat
23	Mitochondria	676463	676472	(T)10	monomeric_repeat
24	Mitochondria	677393	677403	(A)11	monomeric_repeat
25	Mitochondria	680051	680060	(A)10	monomeric_repeat
26	Mitochondria	42842	42853	(TG)6	dinucleotide_repeat
27	Mitochondria	82362	82373	(AT)6	dinucleotide_repeat
28	Mitochondria	220411	220422	(AG)6	dinucleotide_repeat
29	Mitochondria	308844	308857	(AG)7	dinucleotide_repeat
30	Mitochondria	358403	358414	(TC)6	dinucleotide_repeat
31	Mitochondria	522223	522236	(AT)7	dinucleotide_repeat
32	Mitochondria	537304	537315	(TA)6	dinucleotide_repeat
33	Mitochondria	597826	597837	(CT)6	dinucleotide_repeat
34	Mitochondria	85596	85613	(GAA)6	trinucleotide_repeat
35	Mitochondria	242832	242846	(TTC)5	trinucleotide_repeat
36	Mitochondria	447601	447615	(TAA)5	trinucleotide_repeat

Table S3. Table S3. Long repeats in the mitochondrial genome of *T. zeylanicus*

Size	Repeat type	Start position in the genome
167	F	402423
138	P	395469
117	F	666459
117	P	676808
100	P	522788
83	P	293751
79	F	471582
78	P	392473
74	F	473027
73	F	662408
70	P	364490
64	P	540746
62	F	189592
61	F	272628
61	P	258212
55	P	540576
53	P	272091
52	P	540659
50	F	643573
48	P	472378
47	F	531722
46	F	695058
46	P	616854
45	F	396448
44	F	351797
44	F	471683
44	P	377537
43	P	290313
42	F	499375
41	F	569494
41	F	378206
41	P	392505
40	P	480921
40	P	397802
40	P	661532
39	F	620182
39	P	480786
38	F	660238
38	P	590945

38	P	395608
37	F	229783
37	F	591560
37	P	701264
37	P	374891
37	P	660484
37	P	683174
36	F	661737
36	F	672627
36	P	409626
36	P	582841
35	P	575766
34	F	485828
34	F	501650
34	F	662821
34	P	523545
34	P	650283
34	P	569536
34	P	487650
33	F	528866
33	F	199251
33	F	601048
33	F	464494
33	F	474271
33	F	526932
33	P	638500
33	P	476537
33	P	660542
32	F	358286
32	F	669665
32	F	596385
31	F	177439
31	F	513453
31	P	338617
31	P	614937
31	P	608559
30	F	389735
30	F	229841
30	F	687867
30	P	234851

Table S4. RNA editing sites in the protein coding genes of *T. zeylanicus* mitochondrial genome

Label	Base	Aa	Triplet pos.	Bases	Codon	Aa change	Effect
atp1	1292	431	2	C→U	CCC→CUC	P→L	hpb to hpb
atp1	1484	495	2	C→U	CCA→CUA	P→L	hpb to hpb
atp1	971	324	2	C→U	UCG→UUG	S→L	hphl to hpb
atp1	1178	393	2	C→U	UCA→UUA	S→L	hphl to hpb
atp4	436	146	1	C→U	CUU→UUU	L→F	hpb to hpb
atp4	56	19	2	C→U	CCA→CUA	P→L	hpb to hpb
atp4	248	83	2	C→U	CCU→CUU	P→L	hpb to hpb
atp4	251	84	2	C→U	CCG→CUG	P→L	hpb to hpb
atp4	76	26	1	C→U	CCG→UCG	P→S	hpb to hphl
atp4	71	24	2	C→U	UCA→UUA	S→L	hphl to hpb
atp4	89	30	2	C→U	UCA→UUA	S→L	hphl to hpb
atp4	215	72	2	C→U	UCG→UUG	S→L	hphl to hpb
atp4	395	132	2	C→U	UCA→UUA	S→L	hphl to hpb
atp4	470	157	2	C→U	UCA→UUA	S→L	hphl to hpb
atp4	416	139	2	C→U	ACU→AUU	T→I	hphl to hpb
atp4	326	109	2	C→U	ACG→AUG	T→M	hphl to hpb
atp6	886	296	1	C→U	CAU→UAU	H→Y	hbhl to hbhl
atp6	571	191	1	C→U	CUU→UUU	L→F	hpb to hpb

atp6	590	197	2	C→U	CCG→CUG	P→L	hpb to hpb
atp6	692	231	2	C→U	CCC→CUC	P→L	hpb to hpb
atp6	460	154	1	C→U	CCA→UCA	P→S	hpb to hphl
atp6	883	295	1	C→U	CCU→UCU	P→S	hpb to hphl
atp6	652	218	1	C→U	CGC→UGC	R→C	hphl to hphl
atp6	470	157	2	C→U	UCU→UUU	S→F	hphl to hpb
atp6	647	216	2	C→U	UCC→UUC	S→F	hphl to hpb
atp6	971	324	2	C→U	UCU→UUU	S→F	hphl to hpb
atp6	539	180	2	C→U	UCA→UUA	S→L	hphl to hpb
atp6	659	220	2	C→U	UCG→UUG	S→L	hphl to hpb
atp6	824	275	2	C→U	UCA→UUA	S→L	hphl to hpb
atp6	908	303	2	C→U	UCA→UUA	S→L	hphl to hpb
atp6	950	317	2	C→U	UCA→UUA	S→L	hphl to hpb
atp6	1130	377	2	C→U	ACA→AUA	T→I	hphl to hpb
atp8	55	19	1	C→U	CUC→UUC	L→F	hpb to hpb
atp8	58	20	1	C→U	CUC→UUC	L→F	hpb to hpb
atp8	403	135	1	C→U	CGG→UGG	R→W	hphl to hpb
atp8	47	16	2	C→U	UCA→UUA	S→L	hphl to hpb
atp8	200	67	2	C→U	UCG→UUG	S→L	hphl to hpb
atp9	224	75	2	C→U	CCA→CUA	P→L	hpb to hpb
atp9	256	86	1	C→U	CGA→UGA	R→*	hphl to stop
atp9	53	18	2	C→U	UCA→UUA	S→L	hphl to hpb
atp9	125	42	2	C→U	UCG→UUG	S→L	hphl to hpb
atp9	167	56	2	C→U	UCA→UUA	S→L	hphl to hpb
atp9	215	72	2	C→U	UCG→UUG	S→L	hphl to hpb
atp9	245	82	2	C→U	UCA→UUA	S→L	hphl to hpb
ccmb	28	10	1	C→U	CAU→UAU	H→Y	hbhl to hbhl
ccmb	149	50	2	C→U	CCG→CUG	P→L	hpb to hpb
ccmb	164	55	2	C→U	CCG→CUG	P→L	hpb to hpb
ccmb	179	60	2	C→U	CCU→CUU	P→L	hpb to hpb
ccmb	188	63	2	C→U	CCU→CUU	P→L	hpb to hpb
ccmb	338	113	2	C→U	CCG→CUG	P→L	hpb to hpb
ccmb	392	131	2	C→U	CCG→CUG	P→L	hpb to hpb
ccmb	407	136	2	C→U	CCG→CUG	P→L	hpb to hpb
ccmb	476	159	2	C→U	CCA→CUA	P→L	hpb to hpb
ccmb	503	168	2	C→U	CCA→CUA	P→L	hpb to hpb
ccmb	572	191	2	C→U	CCG→CUG	P→L	hpb to hpb
ccmb	43	15	1	C→U	CCC→UCC	P→S	hpb to hphl
ccmb	181	61	1	C→U	CCC→UCC	P→S	hpb to hphl
ccmb	304	102	1	C→U	CGU→UGU	R→C	hphl to hphl
ccmb	313	105	1	C→U	CGU→UGU	R→C	hphl to hphl
ccmb	424	142	1	C→U	CGU→UGU	R→C	hphl to hphl
ccmb	154	52	1	C→U	CGG→UGG	R→W	hphl to hpb

ccmb	286	96	1	C→U	CGG→UGG	R→W	hphl to hpb
ccmb	367	123	1	C→U	CGG→UGG	R→W	hphl to hpb
ccmb	512	171	2	C→U	UCC→UUC	S→F	hphl to hpb
ccmb	566	189	2	C→U	UCC→UUC	S→F	hphl to hpb
ccmb	569	190	2	C→U	UCU→UUU	S→F	hphl to hpb
ccmb	71	24	2	C→U	UCA→UUA	S→L	hphl to hpb
ccmb	80	27	2	C→U	UCG→UUG	S→L	hphl to hpb
ccmb	128	43	2	C→U	UCA→UUA	S→L	hphl to hpb
ccmb	428	143	2	C→U	UCG→UUG	S→L	hphl to hpb
ccmb	467	156	2	C→U	UCG→UUG	S→L	hphl to hpb
ccmb	485	162	2	C→U	UCA→UUA	S→L	hphl to hpb
ccmb	494	165	2	C→U	UCA→UUA	S→L	hphl to hpb
ccmb	551	184	2	C→U	UCA→UUA	S→L	hphl to hpb
ccmb	554	185	2	C→U	UCG→UUG	S→L	hphl to hpb
ccmC	179	60	2	C→U	GCG→GUG	A→V	hpb to hpb
ccmC	467	156	2	C→U	GCU→GUU	A→V	hpb to hpb
ccmC	103	35	1	C→U	CAU→UAU	H→Y	hbhl to hbhl
ccmC	400	134	1	C→U	CUU→UUU	L→F	hpb to hpb
ccmC	128	43	2	C→U	CCU→CUU	P→L	hpb to hpb
ccmC	161	54	2	C→U	CCG→CUG	P→L	hpb to hpb
ccmC	236	79	2	C→U	CCA→CUA	P→L	hpb to hpb
ccmC	446	149	2	C→U	CCG→CUG	P→L	hpb to hpb
ccmC	473	158	2	C→U	CCG→CUG	P→L	hpb to hpb
ccmC	575	192	2	C→U	CCC→CUC	P→L	hpb to hpb
ccmC	608	203	2	C→U	CCC→CUC	P→L	hpb to hpb
ccmC	650	217	2	C→U	CCU→CUU	P→L	hpb to hpb
ccmC	656	219	2	C→U	CCA→CUA	P→L	hpb to hpb
ccmC	665	222	2	C→U	CCC→CUC	P→L	hpb to hpb
ccmC	451	151	1	C→U	CCU→UCU	P→S	hpb to hphl
ccmC	499	167	1	C→U	CCA→UCA	P→S	hpb to hphl
ccmC	568	190	1	C→U	CCU→UCU	P→S	hpb to hphl
ccmC	673	225	1	C→U	CCC→UCC	P→S	hpb to hphl
ccmC	262	88	1	C→U	CGU→UGU	R→C	hphl to hphl
ccmC	619	207	1	C→U	CGU→UGU	R→C	hphl to hphl
ccmC	76	26	1	C→U	CGG→UGG	R→W	hphl to hpb
ccmC	184	62	1	C→U	CGG→UGG	R→W	hphl to hpb
ccmC	331	111	1	C→U	CGG→UGG	R→W	hphl to hpb
ccmC	227	76	2	C→U	UCC→UUC	S→F	hphl to hpb
ccmC	299	100	2	C→U	UCU→UUU	S→F	hphl to hpb
ccmC	497	166	2	C→U	UCU→UUU	S→F	hphl to hpb
ccmC	548	183	2	C→U	UCU→UUU	S→F	hphl to hpb
ccmC	605	202	2	C→U	UCC→UUC	S→F	hphl to hpb
ccmC	107	36	2	C→U	UCA→UUA	S→L	hphl to hpb

ccmC	458	153	2	C→U	UCA→UUA	S→L	hphl to hpb
ccmC	521	174	2	C→U	UCG→UUG	S→L	hphl to hpb
ccmC	614	205	2	C→U	UCA→UUA	S→L	hphl to hpb
ccmC	344	115	2	C→U	ACG→AUG	T→M	hphl to hpb
ccmFC	257	86	2	C→U	CCA→CUA	P→L	hpb to hpb
ccmFC	409	137	1	C→U	CGU→UGU	R→C	hphl to hphl
ccmFC	38	13	2	C→U	UCC→UUC	S→F	hphl to hpb
ccmFC	200	67	2	C→U	UCC→UUC	S→F	hphl to hpb
ccmFC	248	83	2	C→U	ACU→AUU	T→I	hphl to hpb
ccmFC	41	14	2	C→U	ACG→AUG	T→M	hphl to hpb
cob	76	26	1	C→U	CAU→UAU	H→Y	hbhl to hbhl
cob	178	60	1	C→U	CAC→UAC	H→Y	hbhl to hbhl
cob	298	100	1	C→U	CAC→UAC	H→Y	hbhl to hbhl
cob	568	190	1	C→U	CAU→UAU	H→Y	hbhl to hbhl
cob	853	285	1	C→U	CAU→UAU	H→Y	hbhl to hbhl
cob	982	328	1	C→U	CAC→UAC	H→Y	hbhl to hbhl
cob	286	96	1	C→U	CUC→UUC	L→F	hpb to hpb
cob	419	140	2	C→U	CCA→CUA	P→L	hpb to hpb
cob	908	303	2	C→U	CCA→CUA	P→L	hpb to hpb
cob	1124	375	2	C→U	CCG→CUG	P→L	hpb to hpb
cob	118	40	1	C→U	CCG→UCG	P→S	hpb to hphl
cob	808	270	1	C→U	CCC→UCC	P→S	hpb to hphl
cob	1081	361	1	C→U	CCU→UCU	P→S	hpb to hphl
cob	358	120	1	C→U	CGG→UGG	R→W	hphl to hpb
cob	47	16	2	C→U	UCC→UUC	S→F	hphl to hpb
cob	680	227	2	C→U	UCU→UUU	S→F	hphl to hpb
cob	737	246	2	C→U	UCU→UUU	S→F	hphl to hpb
cob	1160	387	2	C→U	ACG→AUG	T→M	hphl to hpb
cox1	1103	368	2	C→U	GCU→GUU	A→V	hpb to hpb
cox1	746	249	2	C→U	CCC→CUC	P→L	hpb to hpb
cox1	854	285	2	C→U	CCU→CUU	P→L	hpb to hpb
cox1	352	118	1	C→U	CCA→UCA	P→S	hpb to hphl
cox1	1462	488	1	C→U	CCA→UCA	P→S	hpb to hphl
cox1	1489	497	1	C→U	CCA→UCA	P→S	hpb to hphl
cox1	715	239	1	C→U	CGG→UGG	R→W	hphl to hpb
cox1	868	290	1	C→U	CGG→UGG	R→W	hphl to hpb
cox1	197	66	2	C→U	UCU→UUU	S→F	hphl to hpb
cox1	254	85	2	C→U	UCU→UUU	S→F	hphl to hpb
cox1	452	151	2	C→U	UCU→UUU	S→F	hphl to hpb
cox1	515	172	2	C→U	UCC→UUC	S→F	hphl to hpb
cox1	668	223	2	C→U	UCU→UUU	S→F	hphl to hpb
cox1	761	254	2	C→U	UCC→UUC	S→F	hphl to hpb
cox1	299	100	2	C→U	UCA→UUA	S→L	hphl to hpb

cox1	551	184	2	C→U	UCA→UUA	S→L	hphl to hpb
cox1	1433	478	2	C→U	UCA→UUA	S→L	hphl to hpb
cox2	25	9	1	C→U	CUC→UUC	L→F	hpb to hpb
cox2	278	93	2	C→U	CCG→CUG	P→L	hpb to hpb
cox2	461	154	2	C→U	CCA→CUA	P→L	hpb to hpb
cox2	557	186	2	C→U	CCU→CUU	P→L	hpb to hpb
cox2	544	182	1	C→U	CCU→UCU	P→S	hpb to hphl
cox2	721	241	1	C→U	CCU→UCU	P→S	hpb to hphl
cox2	676	226	1	C→U	CGU→UGU	R→C	hphl to hphl
cox2	163	55	1	C→U	CGG→UGG	R→W	hphl to hpb
cox2	253	85	1	C→U	CGG→UGG	R→W	hphl to hpb
cox2	379	127	1	C→U	CGG→UGG	R→W	hphl to hpb
cox2	742	248	1	C→U	CGG→UGG	R→W	hphl to hpb
cox2	71	24	2	C→U	UCU→UUU	S→F	hphl to hpb
cox2	161	54	2	C→U	UCA→UUA	S→L	hphl to hpb
cox2	476	159	2	C→U	UCA→UUA	S→L	hphl to hpb
cox2	581	194	2	C→U	UCA→UUA	S→L	hphl to hpb
cox2	614	205	2	C→U	UCA→UUA	S→L	hphl to hpb
cox2	623	208	2	C→U	ACC→AUC	T→I	hphl to hpb
cox2	443	148	2	C→U	ACG→AUG	T→M	hphl to hpb
mttb	295	99	1	C→U	CAU→UAU	H→Y	hbhl to hbhl
mttb	364	122	1	C→U	CAU→UAU	H→Y	hbhl to hbhl
mttb	361	121	1	C→U	CUC→UUC	L→F	hpb to hpb
mttb	407	136	2	C→U	CCU→CUU	P→L	hpb to hpb
mttb	409	137	1	C→U	CCC→UCC	P→S	hpb to hphl
mttb	379	127	1	C→U	CGC→UGC	R→C	hphl to hphl
mttb	386	129	2	C→U	UCC→UUC	S→F	hphl to hpb
mttb	314	105	2	C→U	UCG→UUG	S→L	hphl to hpb
mttb	440	147	2	C→U	UCA→UUA	S→L	hphl to hpb
nad1	308	103	2	C→U	CCG→CUG	P→L	hpb to hpb
nad1	215	72	2	C→U	UCC→UUC	S→F	hphl to hpb
nad1	167	56	2	C→U	UCG→UUG	S→L	hphl to hpb
nad2	710	237	2	C→U	UCC→UUC	S→F	hphl to hpb
nad3	127	43	1	C→U	CAC→UAC	H→Y	hbhl to hbhl
nad3	47	16	2	C→U	CCG→CUG	P→L	hpb to hpb
nad3	65	22	2	C→U	CCA→CUA	P→L	hpb to hpb
nad3	188	63	2	C→U	CCG→CUG	P→L	hpb to hpb
nad3	218	73	2	C→U	CCG→CUG	P→L	hpb to hpb
nad3	254	85	2	C→U	CCC→CUC	P→L	hpb to hpb
nad3	193	65	1	C→U	CCU→UCU	P→S	hpb to hphl
nad3	250	84	1	C→U	CCU→UCU	P→S	hpb to hphl
nad3	352	118	1	C→U	CGG→UGG	R→W	hphl to hpb
nad3	26	9	2	C→U	UCU→UUU	S→F	hphl to hpb

nad3	140	47	2	C→U	UCC→UUC	S→F	hphl to hpb
nad3	149	50	2	C→U	UCU→UUU	S→F	hphl to hpb
nad3	233	78	2	C→U	UCU→UUU	S→F	hphl to hpb
nad3	236	79	2	C→U	UCU→UUU	S→F	hphl to hpb
nad3	278	93	2	C→U	UCU→UUU	S→F	hphl to hpb
nad3	320	107	2	C→U	UCU→UUU	S→F	hphl to hpb
nad3	8	3	2	C→U	UCG→UUG	S→L	hphl to hpb
nad3	347	116	2	C→U	UCG→UUG	S→L	hphl to hpb
nad4	86	29	2	C→U	CCU→CUU	P→L	hpb to hpb
nad4	197	66	2	C→U	CCA→CUA	P→L	hpb to hpb
nad4	100	34	1	C→U	CCA→UCA	P→S	hpb to hphl
nad4	55	19	1	C→U	CGG→UGG	R→W	hphl to hpb
nad4	41	14	2	C→U	UCU→UUU	S→F	hphl to hpb
nad4	281	94	2	C→U	UCU→UUU	S→F	hphl to hpb
nad4	95	32	2	C→U	UCA→UUA	S→L	hphl to hpb
nad4	110	37	2	C→U	UCA→UUA	S→L	hphl to hpb
nad4	230	77	2	C→U	UCA→UUA	S→L	hphl to hpb
nad5	608	203	2	C→U	GCC→GUC	A→V	hpb to hpb
nad5	689	230	2	C→U	GCU→GUU	A→V	hpb to hpb
nad5	676	226	1	C→U	CUU→UUU	L→F	hpb to hpb
nad5	242	81	2	C→U	CCG→CUG	P→L	hpb to hpb
nad5	374	125	2	C→U	CCA→CUA	P→L	hpb to hpb
nad5	506	169	2	C→U	CCU→CUU	P→L	hpb to hpb
nad5	539	180	2	C→U	CCU→CUU	P→L	hpb to hpb
nad5	1184	395	2	C→U	CCA→CUA	P→L	hpb to hpb
nad5	217	73	1	C→U	CCC→UCC	P→S	hpb to hphl
nad5	835	279	1	C→U	CCA→UCA	P→S	hpb to hphl
nad5	553	185	1	C→U	CGU→UGU	R→C	hphl to hphl
nad5	631	211	1	C→U	CGC→UGC	R→C	hphl to hphl
nad5	721	241	1	C→U	CGG→UGG	R→W	hphl to hpb
nad5	398	133	2	C→U	UCU→UUU	S→F	hphl to hpb
nad5	629	210	2	C→U	UCU→UUU	S→F	hphl to hpb
nad5	548	183	2	C→U	UCG→UUG	S→L	hphl to hpb
nad5	713	238	2	C→U	UCG→UUG	S→L	hphl to hpb
nad5	725	242	2	C→U	UCA→UUA	S→L	hphl to hpb
nad5	494	165	2	C→U	ACA→AUA	T→I	hphl to hpb
nad6	530	177	2	C→U	GCG→GUG	A→V	hpb to hpb
nad6	169	57	1	C→U	CAU→UAU	H→Y	hbhl to hbhl
nad6	379	127	1	C→U	CAU→UAU	H→Y	hbhl to hbhl
nad6	289	97	1	C→U	CUU→UUU	L→F	hpb to hpb
nad6	26	9	2	C→U	CCU→CUU	P→L	hpb to hpb
nad6	95	32	2	C→U	CCA→CUA	P→L	hpb to hpb
nad6	161	54	2	C→U	CCA→CUA	P→L	hpb to hpb

nad6	103	35	1	C→U	CGC→UGC	R→C	hphl to hphl
nad6	146	49	2	C→U	UCC→UUC	S→F	hphl to hpb
nad6	158	53	2	C→U	UCC→UUC	S→F	hphl to hpb
nad6	446	149	2	C→U	UCC→UUC	S→F	hphl to hpb
nad6	569	190	2	C→U	UCU→UUU	S→F	hphl to hpb
nad6	83	28	2	C→U	UCG→UUG	S→L	hphl to hpb
nad6	191	64	2	C→U	UCA→UUA	S→L	hphl to hpb
nad9	190	64	1	C→U	CAU→UAU	H→Y	hbhl to hbhl
nad9	223	75	1	C→U	CAU→UAU	H→Y	hbhl to hbhl
nad9	406	136	1	C→U	CAU→UAU	H→Y	hbhl to hbhl
nad9	113	38	2	C→U	CCA→CUA	P→L	hpb to hpb
nad9	311	104	2	C→U	CCA→CUA	P→L	hpb to hpb
nad9	298	100	1	C→U	CCG→UCG	P→S	hpb to hphl
nad9	328	110	1	C→U	CGG→UGG	R→W	hphl to hpb
nad9	14	5	2	C→U	UCC→UUC	S→F	hphl to hpb
nad9	92	31	2	C→U	UCU→UUU	S→F	hphl to hpb
nad9	356	119	2	C→U	UCU→UUU	S→F	hphl to hpb
nad9	368	123	2	C→U	UCC→UUC	S→F	hphl to hpb
nad9	539	180	2	C→U	UCU→UUU	S→F	hphl to hpb
nad9	167	56	2	C→U	UCG→UUG	S→L	hphl to hpb
nad9	398	133	2	C→U	UCA→UUA	S→L	hphl to hpb
rpl16	232	78	1	C→U	CUC→UUC	L→F	hpb to hpb
rpl16	335	112	2	C→U	CCA→CUA	P→L	hpb to hpb
rpl16	401	134	2	C→U	CCA→CUA	P→L	hpb to hpb
rpl16	124	42	1	C→U	CGU→UGU	R→C	hphl to hphl
rpl16	407	136	2	C→U	UCG→UUG	S→L	hphl to hpb
rpl16	104	35	2	C→U	ACC→AUC	T→I	hphl to hpb
rpl16	164	55	2	C→U	ACA→AUA	T→I	hphl to hpb
rpl2	488	163	2	C→U	GCG→GUG	A→V	hpb to hpb
rpl2	332	111	2	C→U	UCC→UUC	S→F	hphl to hpb
rpl2	371	124	2	C→U	UCC→UUC	S→F	hphl to hpb
rpl5	64	22	1	C→U	CAC→UAC	H→Y	hbhl to hbhl
rpl5	47	16	2	C→U	CCG→CUG	P→L	hpb to hpb
rpl5	59	20	2	C→U	CCG→CUG	P→L	hpb to hpb
rpl5	512	171	2	C→U	CCA→CUA	P→L	hpb to hpb
rpl5	515	172	2	C→U	CCG→CUG	P→L	hpb to hpb
rpl5	7	3	1	C→U	CCA→UCA	P→S	hpb to hphl
rpl5	166	56	1	C→U	CCG→UCG	P→S	hpb to hphl
rpl5	376	126	1	C→U	CCC→UCC	P→S	hpb to hphl
rpl5	169	57	1	C→U	CGC→UGC	R→C	hphl to hphl
rpl5	35	12	2	C→U	UCA→UUA	S→L	hphl to hpb
rpl5	92	31	2	C→U	UCG→UUG	S→L	hphl to hpb
rpl5	329	110	2	C→U	UCG→UUG	S→L	hphl to hpb

rpl5	332	111	2	C→U	ACA→AUA	T→I	hphl to hpb
rps10	16	6	1	C→U	CGC→UGC	R→C	hphl to hphl
rps10	238	80	1	C→U	CGG→UGG	R→W	hphl to hpb
rps12	196	66	1	C→U	CAU→UAU	H→Y	hbhl to hbhl
rps12	104	35	2	C→U	CCG→CUG	P→L	hpb to hpb
rps12	112	38	1	C→U	CCG→UCG	P→S	hpb to hphl
rps12	232	78	1	C→U	CCU→UCU	P→S	hpb to hphl
rps12	284	95	2	C→U	UCC→UUC	S→F	hphl to hpb
rps12	71	24	2	C→U	UCG→UUG	S→L	hphl to hpb
rps12	221	74	2	C→U	UCG→UUG	S→L	hphl to hpb
rps12	269	90	2	C→U	UCG→UUG	S→L	hphl to hpb
rps13	100	34	1	C→U	CGU→UGU	R→C	hphl to hphl
rps13	256	86	1	C→U	CGU→UGU	R→C	hphl to hphl
rps13	271	91	1	C→U	CGU→UGU	R→C	hphl to hphl
rps13	5	2	2	C→U	UCA→UUA	S→L	hphl to hpb
rps13	26	9	2	C→U	UCA→UUA	S→L	hphl to hpb
rps13	56	19	2	C→U	UCA→UUA	S→L	hphl to hpb
rps14	325	109	1	C→U	CCU→UCU	P→S	hpb to hphl
rps14	248	83	2	C→U	UCC→UUC	S→F	hphl to hpb
rps19	221	74	2	C→U	UCU→UUU	S→F	hphl to hpb
rps19	116	39	2	C→U	UCG→UUG	S→L	hphl to hpb
rps3	1571	524	2	C→U	GCU→GUU	A→V	hpb to hpb
rps3	667	223	1	C→U	CAU→UAU	H→Y	hbhl to hbhl
rps3	1028	343	2	C→U	CCA→CUA	P→L	hpb to hpb
rps3	1379	460	2	C→U	CCG→CUG	P→L	hpb to hpb
rps3	1534	512	1	C→U	CGU→UGU	R→C	hphl to hphl
rps3	1612	538	1	C→U	CGG→UGG	R→W	hphl to hpb
rps3	92	31	2	C→U	UCC→UUC	S→F	hphl to hpb
rps3	1580	527	2	C→U	UCU→UUU	S→F	hphl to hpb
rps3	485	162	2	C→U	UCA→UUA	S→L	hphl to hpb
rps3	515	172	2	C→U	UCA→UUA	S→L	hphl to hpb
rps3	710	237	2	C→U	UCG→UUG	S→L	hphl to hpb
rps3	887	296	2	C→U	UCG→UUG	S→L	hphl to hpb
rps3	1598	533	2	C→U	UCA→UUA	S→L	hphl to hpb
rps4	343	115	1	C→U	CAU→UAU	H→Y	hbhl to hbhl
rps4	967	323	1	C→U	CAU→UAU	H→Y	hbhl to hbhl
rps4	229	77	1	C→U	CUU→UUU	L→F	hpb to hpb
rps4	377	126	2	C→U	CCG→CUG	P→L	hpb to hpb
rps4	1043	348	2	C→U	CCA→CUA	P→L	hpb to hpb
rps4	1052	351	2	C→U	CCU→CUU	P→L	hpb to hpb
rps4	76	26	1	C→U	CCA→UCA	P→S	hpb to hphl
rps4	226	76	1	C→U	CCC→UCC	P→S	hpb to hphl
rps4	1057	353	1	C→U	CGG→UGG	R→W	hphl to hpb

rps4	890	297	2	C$\rightarrow$U	UCC$\rightarrow$UUC	S$\rightarrow$F	hphl to hpb
rps4	992	331	2	C$\rightarrow$U	UCU$\rightarrow$UUU	S$\rightarrow$F	hphl to hpb
rps4	206	69	2	C$\rightarrow$U	UCA$\rightarrow$UUA	S$\rightarrow$L	hphl to hpb
rps4	956	319	2	C$\rightarrow$U	UCG$\rightarrow$UUG	S$\rightarrow$L	hphl to hpb
rps4	482	161	2	C$\rightarrow$U	ACC$\rightarrow$AUC	T$\rightarrow$I	hphl to hpb

Table S6. Homologues sequences in mitochondrial and Chloroplast genomes of Trichopus zeylanicus

Genome	Accession number	Start	End	Genome	Accession number	Start	End
Mitochondria	OR830326	1	2100	Chloroplast	NC_044084.1	102679	100365
Mitochondria	OR830326	1	2100	Chloroplast	NC_044084.1	131906	134220
Mitochondria	OR830326	16875	17634	Chloroplast	NC_044084.1	107126	107944
Mitochondria	OR830326	16875	17634	Chloroplast	NC_044084.1	127459	126641
Mitochondria	OR830326	53432	54234	Chloroplast	NC_044084.1	113786	114668
Mitochondria	OR830326	186623	188053	Chloroplast	NC_044084.1	96229	94554
Mitochondria	OR830326	186623	188053	Chloroplast	NC_044084.1	138356	140031
Mitochondria	OR830326	352454	354340	Chloroplast	NC_044084.1	51153	53181
Mitochondria	OR830326	354358	356252	Chloroplast	NC_044084.1	13394	15550
Mitochondria	OR830326	356251	357063	Chloroplast	NC_044084.1	54279	55169
Mitochondria	OR830326	384126	385466	Chloroplast	NC_044084.1	44702	43212
Mitochondria	OR830326	385496	386693	Chloroplast	NC_044084.1	43081	41704
Mitochondria	OR830326	445530	447617	Chloroplast	NC_044084.1	33911	31630
Mitochondria	OR830326	453996	454732	Chloroplast	NC_044084.1	61402	62184
Mitochondria	OR830326	520402	522790	Chloroplast	NC_044084.1	57623	55159
Mitochondria	OR830326	571931	573088	Chloroplast	NC_044084.1	39043	40274
Mitochondria	OR830326	575916	576773	Chloroplast	NC_044084.1	8859	7853
Mitochondria	OR830326	576883	578089	Chloroplast	NC_044084.1	3126	4479
Mitochondria	OR830326	578220	579198	Chloroplast	NC_044084.1	4824	5831
Mitochondria	OR830326	583170	584417	Chloroplast	NC_044084.1	81390	79972
Mitochondria	OR830326	584428	585113	Chloroplast	NC_044084.1	79881	79116
Mitochondria	OR830326	585122	586542	Chloroplast	NC_044084.1	81728	83325
Mitochondria	OR830326	585122	586542	Chloroplast	NC_044084.1	152857	151260
Mitochondria	OR830326	586542	587355	Chloroplast	NC_044084.1	25181	24202
Mitochondria	OR830326	608082	608590	Chloroplast	NC_044084.1	40802	40243
Mitochondria	OR830326	617401	618248	Chloroplast	NC_044084.1	69681	70606

Mitochondria	OR830326	692878	695186	Chloroplast	NC_044084.1	17896	20425
Mitochondria	OR830326	568878	569338	Chloroplast	NC_044084.1	90604	91146
Mitochondria	OR830326	568878	569338	Chloroplast	NC_044084.1	143981	143439
Mitochondria	OR830326	402245	402590	Chloroplast	NC_044084.1	102866	102497
Mitochondria	OR830326	402245	402590	Chloroplast	NC_044084.1	131719	132088
Mitochondria	OR830326	587501	587804	Chloroplast	NC_044084.1	69622	69310
Mitochondria	OR830326	696072	696422	Chloroplast	NC_044084.1	37419	37035
Mitochondria	OR830326	225050	225329	Chloroplast	NC_044084.1	103881	103602
Mitochondria	OR830326	225050	225329	Chloroplast	NC_044084.1	130704	130983
Mitochondria	OR830326	3709	4028	Chloroplast	NC_044084.1	104614	104284
Mitochondria	OR830326	3709	4028	Chloroplast	NC_044084.1	129971	130301
Mitochondria	OR830326	17676	17958	Chloroplast	NC_044084.1	103200	102891
Mitochondria	OR830326	17676	17958	Chloroplast	NC_044084.1	131385	131694
Mitochondria	OR830326	583170	583443	Chloroplast	NC_044084.1	153195	153490
Mitochondria	OR830326	127553	127824	Chloroplast	NC_044084.1	64494	64800
Mitochondria	OR830326	233498	233752	Chloroplast	NC_044084.1	106263	106574
Mitochondria	OR830326	233498	233752	Chloroplast	NC_044084.1	128322	128011
Mitochondria	OR830326	614228	614453	Chloroplast	NC_044084.1	12376	12118
Mitochondria	OR830326	162264	162433	Chloroplast	NC_044084.1	761	584
Mitochondria	OR830326	381254	381377	Chloroplast	NC_044084.1	103476	103353
Mitochondria	OR830326	381254	381377	Chloroplast	NC_044084.1	131109	131232
Mitochondria	OR830326	695193	695329	Chloroplast	NC_044084.1	20662	20795
Mitochondria	OR830326	263521	263664	Chloroplast	NC_044084.1	32401	32554
Mitochondria	OR830326	428943	429060	Chloroplast	NC_044084.1	26345	26462
Mitochondria	OR830326	583067	583170	Chloroplast	NC_044084.1	81583	81686
Mitochondria	OR830326	583067	583170	Chloroplast	NC_044084.1	153002	152899
Mitochondria	OR830326	261424	261546	Chloroplast	NC_044084.1	23844	23704
Mitochondria	OR830326	453206	453290	Chloroplast	NC_044084.1	63848	63764
Mitochondria	OR830326	277425	277541	Chloroplast	NC_044084.1	106810	106692

Mitochondria	OR830326	277425	277541	Chloroplast	NC_044084.1	127775	127893
Mitochondria	OR830326	206254	206332	Chloroplast	NC_044084.1	50855	50933
Mitochondria	OR830326	104227	104288	Chloroplast	NC_044084.1	17458	17397
Mitochondria	OR830326	555870	555960	Chloroplast	NC_044084.1	77486	77378
Mitochondria	OR830326	277343	277446	Chloroplast	NC_044084.1	127647	127750
Mitochondria	OR830326	96635	96713	Chloroplast	NC_044084.1	48844	48926
Mitochondria	OR830326	452954	453024	Chloroplast	NC_044084.1	63996	63927
Mitochondria	OR830326	455269	455332	Chloroplast	NC_044084.1	101898	101844
Mitochondria	OR830326	455269	455332	Chloroplast	NC_044084.1	132687	132741
Mitochondria	OR830326	154754	154797	Chloroplast	NC_044084.1	110265	110308
Mitochondria	OR830326	229757	229817	Chloroplast	NC_044084.1	98498	98560
Mitochondria	OR830326	229757	229817	Chloroplast	NC_044084.1	136087	136025
Mitochondria	OR830326	231544	231594	Chloroplast	NC_044084.1	99924	99974
Mitochondria	OR830326	231544	231594	Chloroplast	NC_044084.1	134661	134611
Mitochondria	OR830326	342989	343029	Chloroplast	NC_044084.1	106569	106529
Mitochondria	OR830326	342989	343029	Chloroplast	NC_044084.1	128016	128056
Mitochondria	OR830326	127503	127551	Chloroplast	NC_044084.1	64400	64457
Mitochondria	OR830326	665712	665761	Chloroplast	NC_044084.1	62017	61968
Mitochondria	OR830326	287403	287433	Chloroplast	NC_044084.1	98519	98489
Mitochondria	OR830326	287403	287433	Chloroplast	NC_044084.1	136066	136096
Mitochondria	OR830326	35949	35978	Chloroplast	NC_044084.1	10060	10089
Mitochondria	OR830326	240670	240702	Chloroplast	NC_044084.1	80528	80560
Mitochondria	OR830326	144753	144782	Chloroplast	NC_044084.1	29695	29724
Mitochondria	OR830326	379505	379531	Chloroplast	NC_044084.1	23725	23751

Table S7. Details of mitochondrial genes used in the phylogenetic study

Species	Name and accession number of the genes					
	atp1	ccmFc	cox2	mttB	nad4	rps12
Oryza sativa	AAZ99242.1	AAZ99255.1	AAZ99237.1	AAZ99251.1	AAZ99271.1	AAZ99246.1
Phoenix dactylifera	YP_005090378.1	YP_005090377.1	YP_005090388.1	YP_005090387.1	YP_005090381.1	YP_005090399.1
Allium cepa	YP_009252184.1	YP_009252186.1	YP_009252409.1	YP_009252191.1	YP_009252193.1	YP_009252181.1
Cocos nucifera	YP_009315990.1	YP_009316006.1	YP_009315988.1	YP_009315946.1	YP_009315969.1	YP_009316003.1
Triticum aestivum	YP_009433705.1	YP_009433720.1	YP_009433727.1	YP_009433732.1	YP_009433721.1	YP_009433734.1
Asparagus officinalis	YP_010021905.1	YP_010021919.1	YP_010021913.1	YP_010021902.1	YP_010021926.1	YP_010021912.1
Pandanus odorifer	YP_010883776.1	YP_010883770.1	YP_010883793.1	YP_010883773.1	YP_010883800.1	YP_010883784.1
Pinellia ternata	YP_010921743.1	YP_010921741.1	YP_010921715.1	YP_010921747.1	YP_010921723.1	YP_010921748.1
Pontederia crassipes	YP_010990211.1	YP_010990207.1	YP_010990206.1	YP_010990219.1	YP_010990212.1	YP_010990201.1
Aletris obovata	QKO27160.1	QKO26509.1	QKO26449.1	QKO27799.1	QKO26925.1	UYP39204.1
Burmannia capitata	QKO27140.1	QKO26492.1	QKO26430.1	QKO27780.1	QKO26907.1	QKO27028.1
Burmannia itoana	ACD71511.1	QKO26493.1	QKO26431.1	QKO27781.1	QKO26908.1	QKO27029.1
Dioscorea cayenensis subsp. rotundata	XP_039120136.1	XP_0391317	XP_039131691.1	XP_039118915.1	XP_039119212.1	XP_039118285.1
Dioscorea membranacea	QKO27102.1	QKO26454.1	QKO26392.1	QKO27742.1	QKO26870.1	QKO26991.1
Haplothismia exannulata	QKO27154.1	QKO26503.1	QKO26444.1	QKO27794.1	QKO26920.1	QKO27038.1
Tacca leontopetaloides	QKO27153.1	QKO26502.1	QKO26443.1	QKO27793.1	QKO26919.1	QKO27037.1
Arabidopsis thaliayana	YP_009472105.2	YP_009472113.2	YP_009472098.2	YP_009472115.2	YP_009472128.2	YP_009472123.2
Brassica oleracea	YP_004927463.1	YP_004927509.1	YP_004927497.1	USF18091.1	YP_004927458.1	YP_004927471.1
